# Shifts in type 2 vomeronasal receptor expression during postnatal development in the lungfish olfactory organ

**DOI:** 10.1111/joa.70129

**Published:** 2026-03-08

**Authors:** Shoko Nakamuta, Zicong Zhang, Masato Nikaido, Takuya Yokoyama, Yoshio Yamamoto, Nobuaki Nakamuta

**Affiliations:** ^1^ Laboratory of Veterinary Anatomy, Faculty of Veterinary Medicine Iwate University Morioka Japan; ^2^ Institute for the Advanced Study of Human Biology Kyoto University Kyoto Japan; ^3^ School of Life Science and Technology Institute of Science Tokyo Tokyo Japan

**Keywords:** evolution, in situ hybridization, lungfish, postnatal development, vomeronasal organ, vomeronasal receptors

## Abstract

Many tetrapods possess two distinct olfactory organs: the olfactory epithelium (OE) and the vomeronasal organ (VNO). Fish have only the OE, but lungfish—the closest living relative of tetrapods among fish— possess a lamellar OE and a primitive VNO called a recess epithelium (RecE). Vomeronasal receptor type 2 (V2R) genes in lungfish can be classified into three categories: those expressed only in the lamellar OE, those expressed only in the RecE, and those expressed in both the lamellar OE and the RecE. In this study, we compared V2R expression patterns in *Protopterus annectens* of different body sizes to examine how expression changes with growth. V2Rs expressed exclusively in the lamellar OE in small individuals remained restricted to the lamellar OE in large individuals, and V2Rs expressed exclusively in the RecE in small individuals also remained RecE‐specific in large individuals. In contrast, among the V2Rs expressed in both the lamellar OE and the RecE in small individuals, some maintained expression in both tissues, while others became restricted to the RecE in large individuals. Medium‐sized individuals showed intermediate expression patterns between small and large specimens. These results suggest that a subset of V2Rs initially expressed in both the lamellar OE and the RecE lose expression in the lamellar OE as the individual matures, becoming restricted to the RecE, and that functional separation between the lamellar OE and the RecE is still incomplete in juveniles and becomes more distinct during growth. These findings might represent the developmental process of the bimodal olfactory system in vertebrates. In the common ancestors of lungfish and tetrapods, there might be no functional separation between the OE and VNO. However, it can be speculated that olfactory functions have partially separated between the OE (lamellar OE) and VNO (RecE) in extant lungfish, while they have completely separated between the OE and VNO in extant tetrapods which acquired more developed VNO.

## Graphical abstract

Lungfish possess a lamellar OE and a primitive VNO called as a recess epithelium (RecE). Among the lungfish V2Rs expressed in both the lamellar OE and the RecE in small individuals, some became restricted to the RecE in large individuals. These results suggest functional separation between the lamellar OE and the RecE is still incomplete in juveniles and it becomes more distinct during growth.

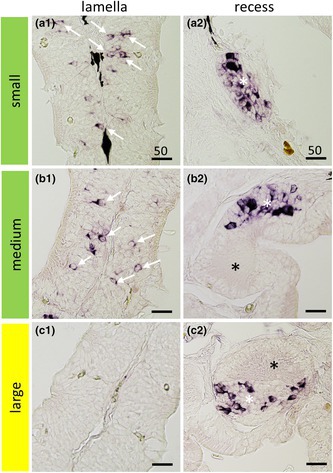

## 1 INTRODUCTION

With a few exceptions (birds, humans, crocodilians, etc.), tetrapods possess two olfactory organs: the olfactory epithelium (OE) and the vomeronasal organ (VNO). It was previously thought that the OE detects general odorants while the VNO detects pheromones. However, it is now believed that their olfactory functions are partially overlapping (Baum, 2012; Suárez et al., 2012; Vargas‐Barroso et al., 2017). In mammals, the OE contains ciliated olfactory receptor cells (ORCs), and the VNO contains microvillous ORCs (Menco, 1997). Axons from the OE project to the main olfactory bulb, whereas those from the VNO project to the accessory olfactory bulb (Buck, 2000). Fish, on the other hand, lack the VNO, and both ciliated and microvillous ORCs are distributed in the fish OE (Taniguchi & Taniguchi, 2014).

The major olfactory receptors in vertebrates include odorant receptors (ORs), trace amine‐associated receptors (TAARs), and vomeronasal receptors type 1 and type 2 (V1Rs and V2Rs). In mammals, ORs and TAARs are expressed in the OE, while V1Rs and V2Rs are expressed in the VNO (Poncelet & Shimeld, 2020). In fish lacking the VNO, all of these olfactory receptors are expressed in the OE: ciliated ORCs express ORs or TAARs, whereas microvillous ORCs express V1Rs or V2Rs (Hansen et al., 2004). In addition to the relationship between olfactory receptor expression and the fine structure of ORCs, the association between olfactory receptors and olfactory signal transduction molecules is conserved across many vertebrates, from fish to mammals (Hansen et al., 2004; Poncelet & Shimeld, 2020). Regarding G proteins and ion channels involved in olfactory signal transduction: ORs and TAARs co‐express with Gαolf and cyclic nucleotide‐gated channel alpha 2 (Brunet et al., 1996; Dewan, 2021), V1Rs with Gαi2 and transient receptor potential channel 2 (TRPC2), and V2Rs with Gαo and TRPC2 (Herrada & Dulac, 1997; Liman et al., 1999).

The lungfish belongs to the lobe‐finned fishes and is the closest living relative of tetrapods. Several research groups, including ours, have reported that the lungfish possesses a primitive VNO in addition to the OE (González et al., 2010; Kim & Park, 2021; Nakamuta et al., 2012; Wittmer & Nowack, 2017). This supports the hypothesis that the VNO arose during the evolutionary transition from fish to tetrapods in vertebrate phylogeny (Hansen et al., 2004; Taniguchi & Taniguchi, 2014). The nasal sac of the lungfish contains lamellae arranged on both sides of the midline raphe, with recesses located at the base of the lamellae. The lamellae contain lamellar OE, while the recesses contain recess epithelium (RecE)—two distinct types of sensory epithelium with different properties. The recess is also referred to as an epithelial crypt (González et al., 2010; Wittmer & Nowack, 2017) or a vomero‐like epithelial crypt (Kim & Park, 2021), while the RecE is also termed crypt sensory epithelium (Kim & Park, 2021; Wittmer & Nowack, 2017). Based on the fine structure of ORCs and their projection patterns to the olfactory bulb, lamellar OE is thought to correspond to the OE of ray‐finned fish, while RecE corresponds to the VNO of tetrapods (González et al., 2010; Nakamuta et al., 2012). The expression of olfactory receptors, G proteins, and signaling molecules further supports this hypothesis (Nakamuta, Sakuma, et al., 2023; Nakamuta et al., 2023a, 2023b; Nakamuta et al., 2024).

The recess containing RecE in the olfactory organ of lungfish is located at the base of the lamellae, and its morphology differs markedly from the tetrapod VNO, which has a pouch or tubular structure independent of the OE. In African and South American lungfish, large individuals are known to possess more recesses than smaller ones (Nakamuta et al., 2022; Wittmer & Nowack, 2017). This suggests that lungfish recesses are formed repeatedly after birth, unlike the tetrapod VNO, which develops only once during embryogenesis.

Most cells expressing V1Rs in the lungfish olfactory organ are localized in the lamellar OE, with only a few found in the RecE (Nakamuta, Sakuma, et al., 2023; Nakamuta et al., 2023a, 2023b). Moreover, the distribution of V1R‐expressing cells appears to change with growth: juveniles show a stronger tendency for V1R cells to localize in the lamellar OE rather than in the RecE, whereas this tendency is less pronounced in adults (Nakamuta et al., 2023b).

We recently demonstrated, through analysis of V2R expression in the olfactory organs of the African lungfish *Protopterus annectens*, that lungfish V2R genes can be classified into three categories based on their expression sites: those expressed only in the lamellar OE, those expressed only in the RecE, and those expressed in both the lamellar OE and RecE (Nakamuta et al., 2024). In the present study, we analyzed V2R expression sites in *P. annectens* of various sizes and obtained results suggesting that the expression sites of some V2Rs shift from the lamellar OE to the RecE as individuals grow. Here, we discuss the significance of this shift in V2R expression in lungfish during growth.

## 2 MATERIALS AND METHODS

### 2.1 Animals

The study protocol was approved by the local animal ethics committee of Iwate University. African lungfishes, *P. annectens*, were obtained from commercial suppliers. The six specimens analyzed in this study were divided into three categories based on body length: small (<20 cm, #1 and #2), medium (20–35 cm, #3 and #4), and large (>35 cm, #5 and #6). Detailed information on the specimens is provided in Table 1. Prior to dissection, the fish were anesthetized with tricaine methanesulfonate (MS‐222) and euthanized by decapitation. The olfactory organs were dissected from the heads and fixed in 4% paraformaldehyde in 0.1 M phosphate buffer (pH 7.4). The specimens were then cryoprotected in a sucrose gradient (15%–30% in 0.1 M phosphate buffer), embedded in O.C.T. compound (Sakura Finetek, Tokyo, Japan), and sectioned sagittally using a cryostat. Sections (20 μm thick) were thaw‐mounted onto MAS‐coated slides (Matsunami, Osaka, Japan), air‐dried, and processed for hematoxylin and eosin staining or in situ hybridization.

**TABLE 1:** Animals.

Animal No.	Body length (cm)	Weight (g)	Sex
1	14	8.5	Unknown
2	15.5	11.5	Unknown
3	24	46.9	Female
4	24	47.8	Male
5	36.8	221	Female
6	37	240.2	Male

### 2.2 In situ hybridization

Digoxigenin (DIG)‐labeled probes against G proteins were prepared as described previously (Nakamuta, Sakuma, et al., 2023). DIG‐labeled probes against 20 V2R genes, selected from the 123 V2R genes identified in our earlier study (Nakamuta et al., 2024), were also prepared as described previously (Nakamuta et al., 2024). Probe names and their corresponding target V2R genes (Data S1) examined by in situ hybridization are listed in Table 2.

**TABLE 2:** Number of positive cells labeled by V2R probes in the lamellae and recesses of small, medium, and large *Protopterus annectens*.

Probe	Target V2R gene	Small (<BL20 cm)				Medium (BL20–35 cm)				Large (>BL35 cm)			
		1		2		3		4		5		6	
		Number of positive cells		Number of positive cells		Number of positive cells		Number of positive cells		Number of positive cells		Number of positive cells	
		Lamella	Recess	Lamella	Recess	Lamella	Recess	Lamella	Recess	Lamella	Recess	Lamella	Recess
PA40	annecV2R63	5	0	9	0	3	0	14	0	11	0	7	0
PA41	annecV2R114	2	0	9	0	2	0	10	0	7	0	5	0
PA42	annecV2R120	8	0	8	0	4	0	9	0	5	0	3	0
PA43	annecV2R217	0	106	5	71	0	68	0	19	0	42	0	80
PA45	annecV2R276	3	0	11	0	5	0	11	0	22	0	2	0
PA46	annecV2R392	25	1	16	0	26	0	24	0	30	0	24	0
PA47	annecV2R467	17	0	21	0	26	0	23	0	17	0	17	0
PA48	annecV2R488	5	0	12	0	13	0	13	1	11	0	7	2
PA49	annecV2R526	7	0	15	0	19	0	15	0	36	0	4	0
PA50	annecV2R535	9	0	14	0	18	0	28	0	26	0	18	0
PA51	annecV2R709	15	0	16	0	16	0	15	0	28	0	16	0
PA52	annecV2R755	1	0	3	0	3	0	3	0	2	0	1	0
PA81	annecV2R183	41	22	47	10	60	33	59	10	77	17	24	12
PA82	annecV2R201/202/205/206/208/211/213/214	2	60	14	46	27	62	0	81	0	181	0	124
PA83	annecV2R244	22	69	45	65	67	53	30	29	0	189	0	183
PA84	annecV2R352/353/354/350	4	45	20	46	15	69	0	75	0	131	0	125
PA85	annecV2R357/358/360/363	39	6	31	4	34	1	51	8	34	4	27	3
PA86	annecV2R528	0	21	0	15	0	23	0	37	0	39	0	34
PA87	annecV2R701	15	5	15	7	52	5	15	4	54	5	40	12
PA88	annecV2R712	61	0	23	3	78	3	40	1	115	4	20	2

*Note*: V2Rs expressed only in the lamellar OE are highlighted in blue, only in the RecE in yellow, and in both the lamellar OE and the RecE in green.

Abbreviations: BL, body length; OE, olfactory epithelium; RecE, recess epithelium.

In situ hybridization was performed following the protocol described previously (Nakamuta et al., 2023b). Briefly, sections of the *P. annectens* olfactory organ were hybridized with probes overnight at 55°C, then treated with an alkaline phosphatase‐conjugated anti‐DIG antibody (Roche Diagnostics, Basel, Switzerland), followed by NBT/BCIP stock solution (Roche Diagnostics) for signal detection.

## 3 RESULTS

**FIGURE 1:**
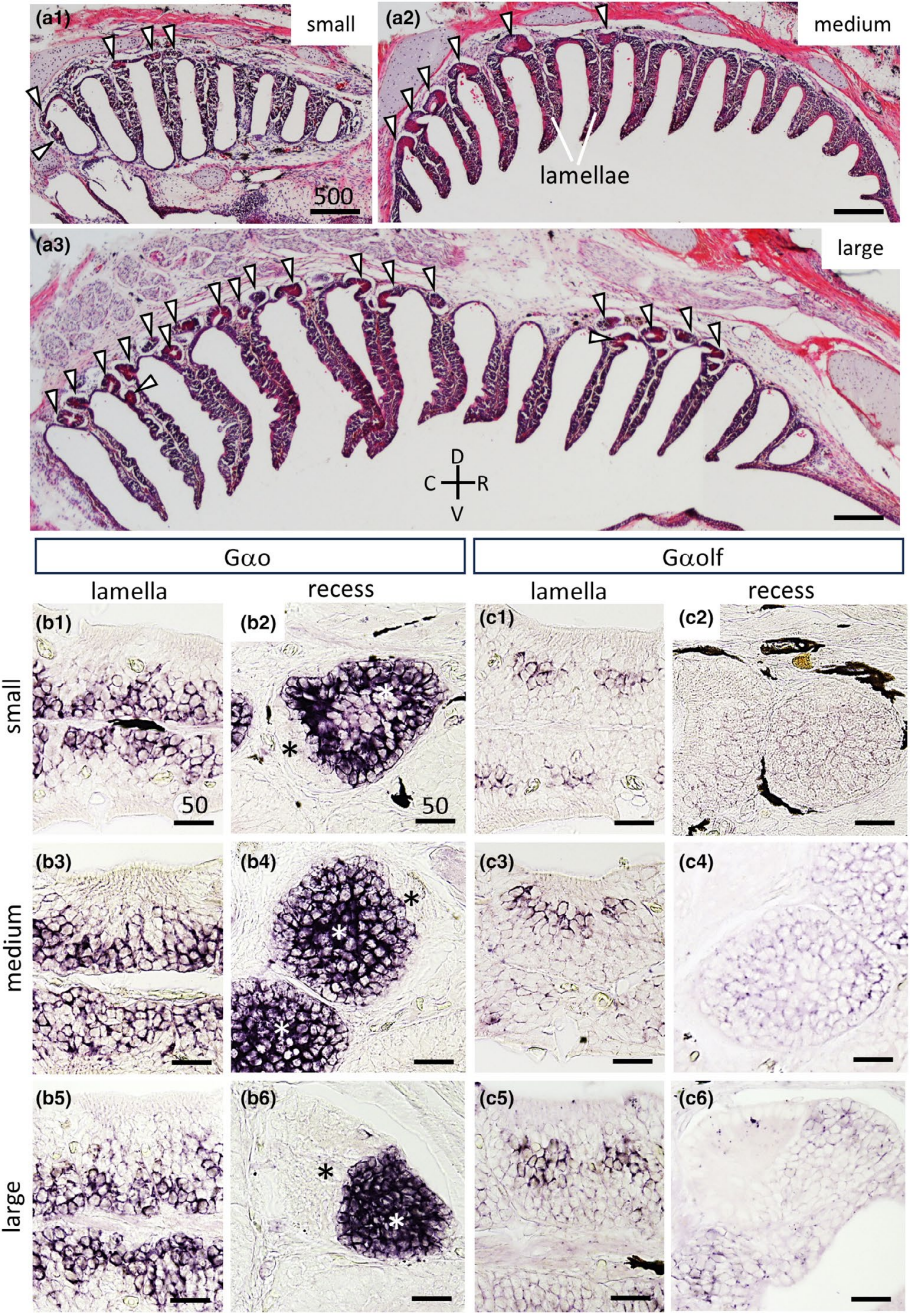
Structure of olfactory organ in the small, medium, and large *Protopterus annectens*. (a1–a3) Hematoxylin and eosin‐stained sagittal sections of olfactory organs in small (body length (BL)14 cm, animal #1), medium (BL 24 cm, #3), and large (BL 37 cm, #6) specimens. Dorsal is top and rostral is right. A number of lamellae are suspended from the dorsal wall, and recesses are located at the base of lamellae (arrowheads). (b1–b6) Gαo expression in the lamellae and recesses of the small, medium, and large specimens. In all specimens, Gαo is expressed in the lower layer of the lamellar OE and in most olfactory receptor cells in the RecE (white asterisks). Black asterisks indicate the glandular epithelium in the recesses. (c1–c6) Gαolf expression in the lamellae and recesses of the small, medium, and large specimens. In all specimens, Gαolf is expressed in the upper layer of the lamellar OE, but not in the RecE. Note that larger specimens have larger olfactory organs with more numerous lamellae and recesses, but the thickness of the lamellae, the diameter of the recesses, and the expression of G proteins are the same among small, medium and large specimens. Scale bars: 500 μm in a1–a3; 50 μm in b1–b6 and c1–c6.

Hematoxylin‐ and eosin‐stained images and G protein expression in the olfactory organs of small, medium, and large *P. annectens* are shown in Figure 1. As reported in previous studies (Nakamuta et al., 2022; Wittmer & Nowack, 2017), larger individuals possessed larger olfactory organs and a greater number of lamellae and recesses (Figure 1a1–a3). However, the thickness of the lamellar OE (~ 200 μm) and the diameter of the recesses (~ 200 μm) did not differ among small, medium, and large individuals. Similarly, the expression of Gαo and Gαolf did not vary with body size. In all specimens, Gαo was expressed in the lower layer of the lamellar OE and in most ORCs of the RecE, whereas Gαolf was expressed in the upper layer of the lamellar OE but not in the RecE (Figure 1b1–b6, c1–c6). The expression of these G proteins in the lamellar OE did not differ according to distance from the midline raphe (proximal, intermediate, or distal regions) of the nasal sac (Figure S1).

After in situ hybridization, the number of positive cells in lamellae and recesses was counted for each specimen: in a single section for medium and large individuals (#3–#6), and across several sections for small individuals (#1–#2). Counts were standardized to ~ 10–20 rows of lamellae or 10–20 recesses (Table 2). However, for the expression analysis of PA82‐84 in the large individuals (#5‐#6), three sections with different distances from the midline raphe of the nasal sac (proximal, intermediate, and distal regions) were used to ensure reliable classification of the expression type. These sections collectively contained ~ 40–50 recesses and 30–40 lamellae.

As demonstrated in our previous study (Nakamuta et al., 2024), *P. annectens* V2Rs were classified into three categories based on their expression patterns: those expressed exclusively in the lamellar OE, those expressed exclusively in the RecE, and those expressed in both the lamellar OE and the RecE (Table 2; Figures S2–S4). Furthermore, we observed differences in V2R expression patterns among individuals of different body sizes.

In the small specimens (#1 and #2), approximately half of the examined V2Rs were expressed exclusively in the lamellar OE (blue in Table 2). Most of the remaining V2Rs were expressed in both the lamellar OE and the RecE (green in Table 2), while only two V2Rs, PA43 and PA86, were expressed exclusively in the RecE (yellow in Table 2). However, for PA43, PA46, and PA88, expression patterns differed between individuals #1 and #2: PA43 was expressed either exclusively in the RecE or in both the lamellar OE and the RecE, whereas PA46 and PA88 were expressed either exclusively in the lamellar OE or in both the lamellar OE and the RecE (Table 2).

V2Rs that were expressed exclusively in the lamellar OE in the small specimens were also expressed exclusively in the lamellar OE in the medium and large specimens (#3–#6) (Table 2; Figure S2). Similarly, V2Rs that were expressed exclusively in the RecE in the small specimens were also expressed exclusively in the RecE in the medium and large specimens (Table 2; Figure S3).

**FIGURE 2:**
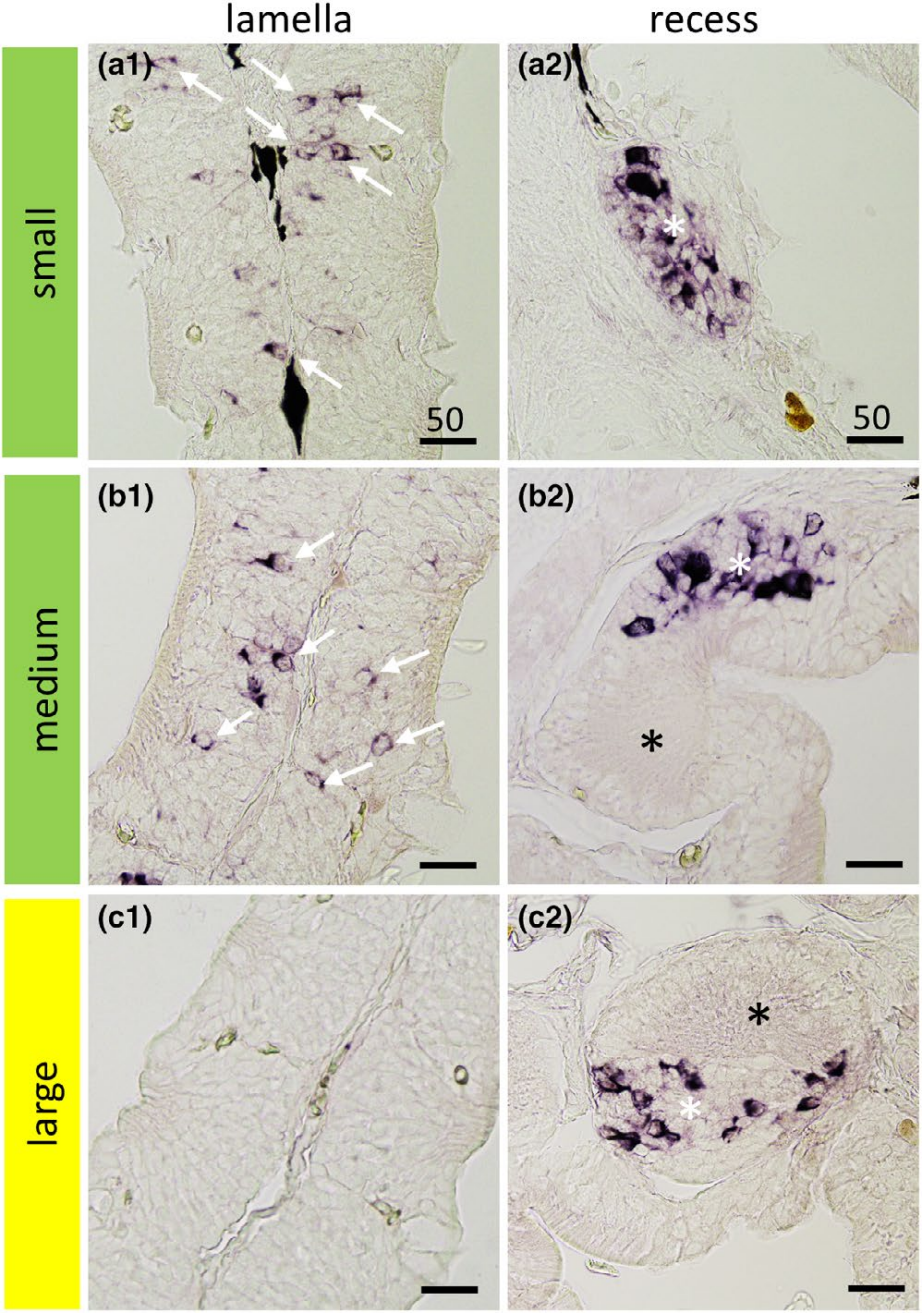
Expression of PA83 in the small, medium, and large *Protopterus annectens*. In the small (a1–a2) and medium (b1–b2) specimens, PA83 is expressed both in the lamellar OE (arrows in a1 and b1) and the RecE (white asterisks in a2 and b2). On the other hand, in the large specimen (c1–c2), PA83 is not expressed in the lamellar OE (c1) but is expressed exclusively in the RecE (white asterisk in c2). Black asterisks indicate the glandular epithelium in the recesses. Scale bars: 50 μm.

In the medium and large specimens (#3–#6), four (PA81, PA85, PA87, and PA88) of the seven V2Rs (PA81–85, PA87–88) that were expressed in both the lamellar OE and the RecE in the small specimens were also expressed in both the lamellar OE and the RecE (Table 2; Figure S4). However, the remaining three, PA82–84, were expressed exclusively in the RecE in the large specimens (#5 and #6). In the medium specimens (#3 and #4), PA83 was expressed in both the lamellar OE and the RecE, as in the small specimens. Expression of PA83 in the small, medium, and large specimens is shown in Figure 2. The expression of PA82 and PA84, however, differed between the two medium specimens: in one specimen (#3, female), as in the small specimens, PA82 and PA84 were expressed in both the lamellar OE and the RecE, whereas in the other specimen (#4, male), as in the large specimens, they were expressed exclusively in the RecE. It cannot be ruled out that the possibility that the difference in expression of PA82 and PA84 between #3 and #4 is derived from the sex difference, though its verification is difficult due to the small sample size. However, this possibility might be considered unlikely, since there was no difference in the expression of PA82 and PA84 between the two large specimens, #5 (female) and #6 (male). Rather, it seems that the V2R expression pattern in the medium specimens displayed intermediate characteristics between those of the small and large specimens.

Finally, to investigate whether recess function varies with body size, we calculated the number of positive cells per one recess (number of positive cells/number of recesses analyzed) for nine V2R probes (PA43, PA81‐PA88), which are the RecE type (V2Rs expressed exclusively in the RecE or expressed in both the lamellar OE and the RecE) (Table 3). Furthermore, the total number of these positive cells was considered the total number of V2R cells per one recess, and the relative abundance of positive cells in total V2R cells per one recess was calculated (Table 3). There were no major differences among these specimens in the number of positive cells for each V2R per one recess or in their relative abundance per one recess, suggesting that body growth does not cause major changes in the function of recess.

**TABLE 3:** Number of V2R positive cells per one recess and relative abundance.

	Small				Medium				Large			
	#1		#2		#3		#4		#5		#6	
	A	B	A	B	A	B	A	B	A	B	A	B
PA43	8.83	25.07	6.45	22.04	11.33	31.95	2.38	13.66	3.82	19.77	4.21	26.37
PA81	2.20	6.24	1.67	5.69	3.67	10.34	0.63	3.60	0.77	4.00	0.44	2.78
PA82	6.67	18.92	5.75	19.63	6.89	19.42	5.06	29.12	4.31	22.32	2.64	16.52
PA83	8.63	24.47	7.22	24.66	4.82	13.58	1.81	10.43	4.61	23.87	3.89	24.38
PA84	5.63	15.96	5.75	19.63	6.27	17.69	4.69	26.96	3.36	17.40	2.84	17.79
PA85	0.60	1.70	0.44	1.52	0.08	0.23	0.44	2.56	0.18	0.94	0.12	0.75
PA86	2.33	6.62	1.36	4.66	1.77	4.99	2.06	11.82	1.77	9.18	1.31	8.19
PA87	0.36	1.01	0.47	1.59	0.38	1.08	0.25	1.44	0.26	1.36	0.44	2.78
PA88	0.00	0.00	0.17	0.57	0.25	0.70	0.07	0.41	0.22	1.15	0.07	0.43
Sum	35.24	100.00	29.28	100.00	35.47	100.00	17.38	100.00	19.31	100.00	15.97	100.00

*Note*: A: Number of positive cells for each V2R per one recess. B: Percentage of positive cells for each V2R in total V2R cells per one recess.

## 4 DISCUSSION

The V2R genes of *P. annectens* are categorized into three groups based on their expression sites: those expressed only in the lamellar OE, those expressed only in the RecE, and those expressed in both the lamellar OE and the RecE. This study revealed that some V2R genes exhibit different expression sites between small and large individuals. Furthermore, the expression sites of three V2R genes (PA82, PA83, and PA84) in medium individuals were intermediate between those in the small and large individuals. These results suggest that certain V2Rs (PA82–84), which are expressed in both the lamellar OE and the RecE in juveniles, lose expression in the lamellar OE as the individual grows and become restricted to the RecE in adults. This finding may represent the process by which the V2R expression became restricted to the VNO during the evolution from teleosts to tetrapods. It can be speculated that V2Rs were expressed both in the OE and VNO in the ancestors of tetrapods, but may have lost expression in the OE during evolution, leading to the current situation where V2Rs are expressed exclusively in the VNO of extant tetrapods.

Changes in V2R expression sites during individual growth have also been observed in amphibians. The olfactory organs of the African clawed frog consist of the main OE and the VNO in tadpoles and of the main OE, middle chamber epithelium (MCE), and VNO in adults (Weiss et al., 2021). During the larval stage, V2R genes are classified as those expressed only in the main OE and those expressed only in the VNO. However, as the main OE—serving as the “water nose”—is reconstructed into the “air nose” during metamorphosis from larva to adult, and a new “water nose,” the MCE, is formed, the V2Rs that were specifically expressed in the main OE shift their expression to the MCE (Syed et al., 2013, 2017). Thus, the shift in V2R expression in the clawed frog reflects the adult MCE taking over the olfactory function previously performed by the larval main OE (Syed et al., 2017). This developmental transition is entirely distinct from the loss of certain V2R expressions in the lamellar OE observed in lungfish juveniles as they mature, as suggested by this study.

Why do some V2Rs (PA82–84) change their expression sites as individuals grow? As the lungfish grows, the number of recesses within the olfactory organ increases and their distribution area expands. Specifically, juvenile recesses are fewer in number and concentrated in the caudomedial region of the olfactory organ, whereas adult recesses are more numerous and distributed throughout the entire organ (Nakamuta et al., 2022; Wittmer & Nowack, 2017). V2Rs expressed only in the RecE are likely to be inefficient in olfactory reception during the juvenile stage because they are expressed in fewer ORCs and occupy a limited area of the olfactory organ. By contrast, V2Rs expressed in both the RecE and the lamellar OE are thought to enable more efficient odorant detection in juveniles, which possess only a few recesses. As growth progresses, however, V2Rs that lose expression in the lamellar OE and become restricted to the RecE may no longer require expression in the lamellar OE, since the increasing number and broader distribution of recesses likely enhance their functional efficiency.

In contrast to PA82–84, PA43 and PA86 were expressed exclusively in the RecE regardless of body size. In juveniles, given the limited number and restricted distribution of recesses, olfactory reception mediated by V2Rs expressed only in the RecE is presumably inefficient. Nevertheless, the fact that PA43 and PA86 are expressed solely in the RecE, even in juveniles, suggests that the olfactory information they mediate may not be particularly important during the juvenile stage. Instead, these V2Rs may be involved in olfactory reception crucial for adults. Olfactory function can vary depending on an individual's habitat, diet, reproductive condition, and social status (Nikonov et al., 2017; Weiss et al., 2021). The habitat and diet of lungfish differ between juveniles and adults: juveniles inhabit lagoons and feed on insects, small mollusks, and crustaceans, whereas adults inhabit open, deeper waters away from the shore and primarily prey on fish (Jorgensen & Joss, 2010). It is therefore likely that PA43 and PA86 detect odorants associated with the habitat, diet, or reproductive state specific to adult lungfish. There is one possibility that the presence of V2Rs which detect odorants specific to the adult lungfish's lifestyle have promoted the separational expression of V2Rs between the OE and the RecE.

In the lungfish olfactory organ, Gαolf, which co‐expresses with ORs and TAARs, is expressed in the lamellar OE in both juveniles and adults, but not in the RecE (Figure 1). Furthermore, in both life stages, most cells expressing V1Rs are localized in the lamellar OE (Nakamuta et al., 2023b). These findings indicate that functional differentiation between the lamellar OE and the RecE is already established in juveniles. Meanwhile, as suggested in this study, some V2Rs that are expressed in both the lamellar OE and the RecE in juveniles lose expression in the lamellar OE as individuals grow, continuing to be expressed only in the RecE. This suggests that while functional separation between the lamellar OE and the RecE is still incomplete in juveniles—with fewer recesses—it becomes more distinct as the number of recesses increases during growth. These findings might represent the developmental process of the bimodal olfactory system in vertebrates. In the common ancestors of lungfish and tetrapods, there might be no functional separation between the OE and VNO. However, it can be speculated that olfactory functions have partially separated between the OE (lamellar OE) and VNO (RecE) in extant lungfish, while they have completely separated between the OE and VNO in extant tetrapods which acquired more developed VNO.

Taken together, the results of this study suggest that among the three types of lungfish V2Rs, classified by expression site, those expressed only in the lamellar OE and those expressed only in the RecE maintain their respective expression sites throughout development. However, among V2Rs expressed in both the lamellar OE and the RecE, some lose expression in the lamellar OE during development and subsequently become restricted to the RecE. This indicates that functional separation between the lamellar OE and the RecE is initially incomplete but becomes progressively more defined as the individual matures. The mechanisms underlying the division of V2R expression into RecE and non‐RecE types, as well as the molecular basis for the developmental loss of V2R expression in the lamellar OE, remain unclear. It can be hypothesized that a transcription factor exists that is exclusively expressed in the RecE and suppresses the expression of some V2Rs. If so, it can be inferred that the target V2Rs of this factor would be the non‐RecE type (V2Rs expressed exclusively in the lamellar OE), while other non‐target V2Rs would be the RecE type (V2Rs expressed exclusively in the RecE, or expressed in both the lamellar OE and the RecE). Since classification of these two types (RecE type and non‐RecE type) does not change with body size, it is thought that the expression of factors involved in this classification remains constant throughout life. On the other hand, the expression shift of some V2Rs—which are expressed in both the lamellae OE and the RecE in juveniles but expressed exclusively in the RecE in adults—can be hypothesized to occur because of a factor suppressing the expression of these V2Rs in the lamellar OE, and this suppression is enhanced with body growth. Epigenetic modulation may be involved in the enhanced suppression of V2R expression during body growth. Further investigations are needed to verify these hypotheses.

## AUTHOR CONTRIBUTIONS

N.N. supervised the project. S.N. conducted animal experiments. S.N., Z.Z., M.N., and N.N. conducted data analysis and wrote the paper. Y.Y. and T.Y. conducted manuscript editing. M.N. and N.N conducted experimental design. All authors read and approved the final manuscript.

## Supporting information


Data S1.

Data S2.

## Data Availability

The data that supports the findings of this study are available in the Supporting Information of this article.
