# Supplementary material for: Shifts in type 2 vomeronasal receptor expression during postnatal development in the lungfish olfactory organ
Source: J Anat. 2026 Mar 8;249(4):978–86. doi: 10.1111/joa.70129 (PMC13398680; doi:10.1111/joa.70129)
Supplement: Supplementary file 1 [file JOA-249-978-s001.pdf]

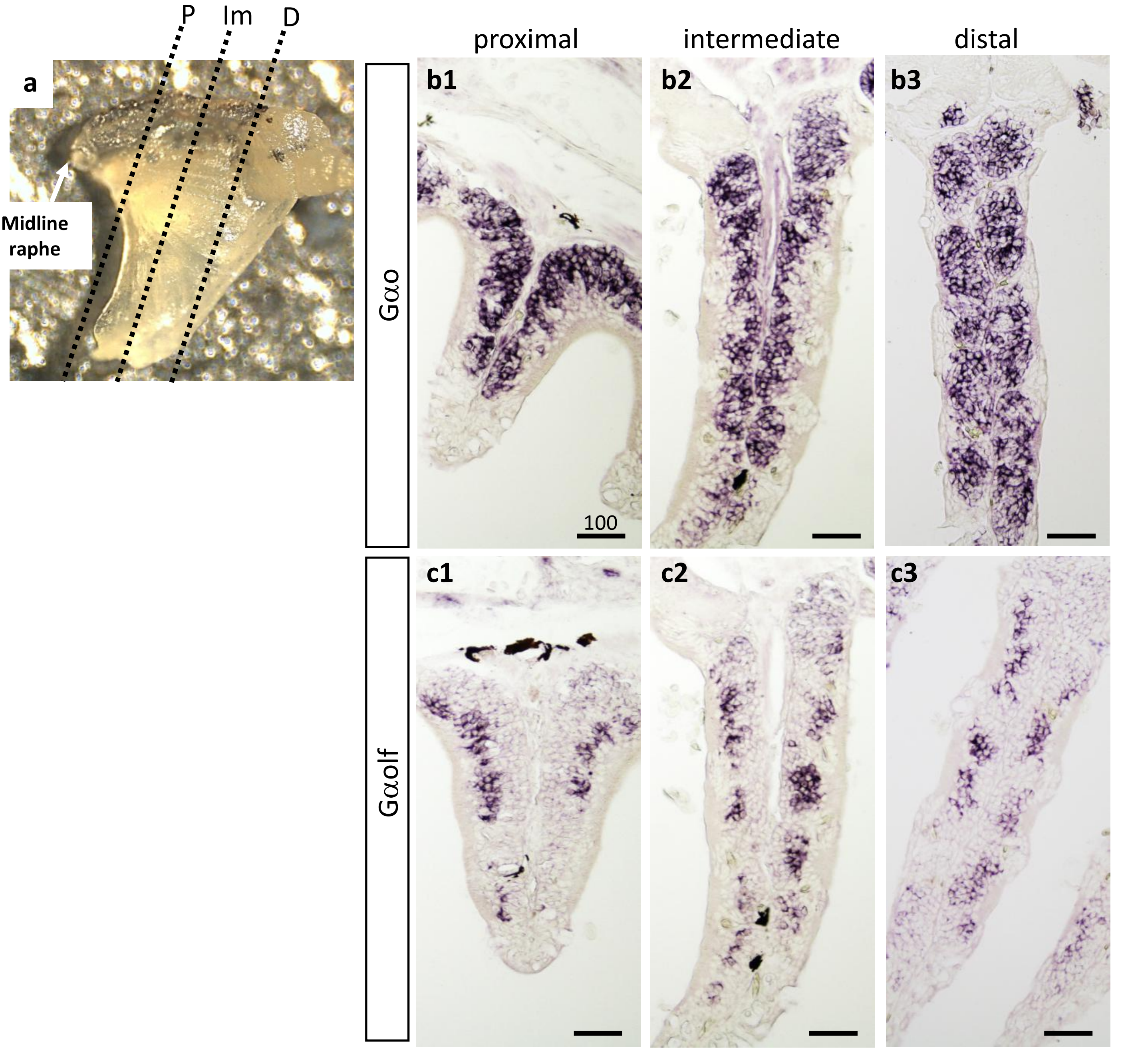


**FIGURE S1** Expression of G proteins in the lamellar OE at the proximal, intermediate and distal regions from the midline raphe of the nasal sac. (a) A lamella cut out from the olfactory organ of *P. annectens*. The sagittal sections of the lamellae cut at line P (proximal region from the midline raphe), Im (intermediate region), and D(distal region) are shown in b1-c1, b2-c2, and b3-c3. (b1-b3, c1-c3) The expression of Gαo and Gαolf in the lamellar OE does not differ based on distance from the midline raphe (proximal, intermediate or distal regions). Gαo is expressed in the lower layer, and Gαolf is in the upper layer of the lamellar OE. Scale bars: 100 μm.

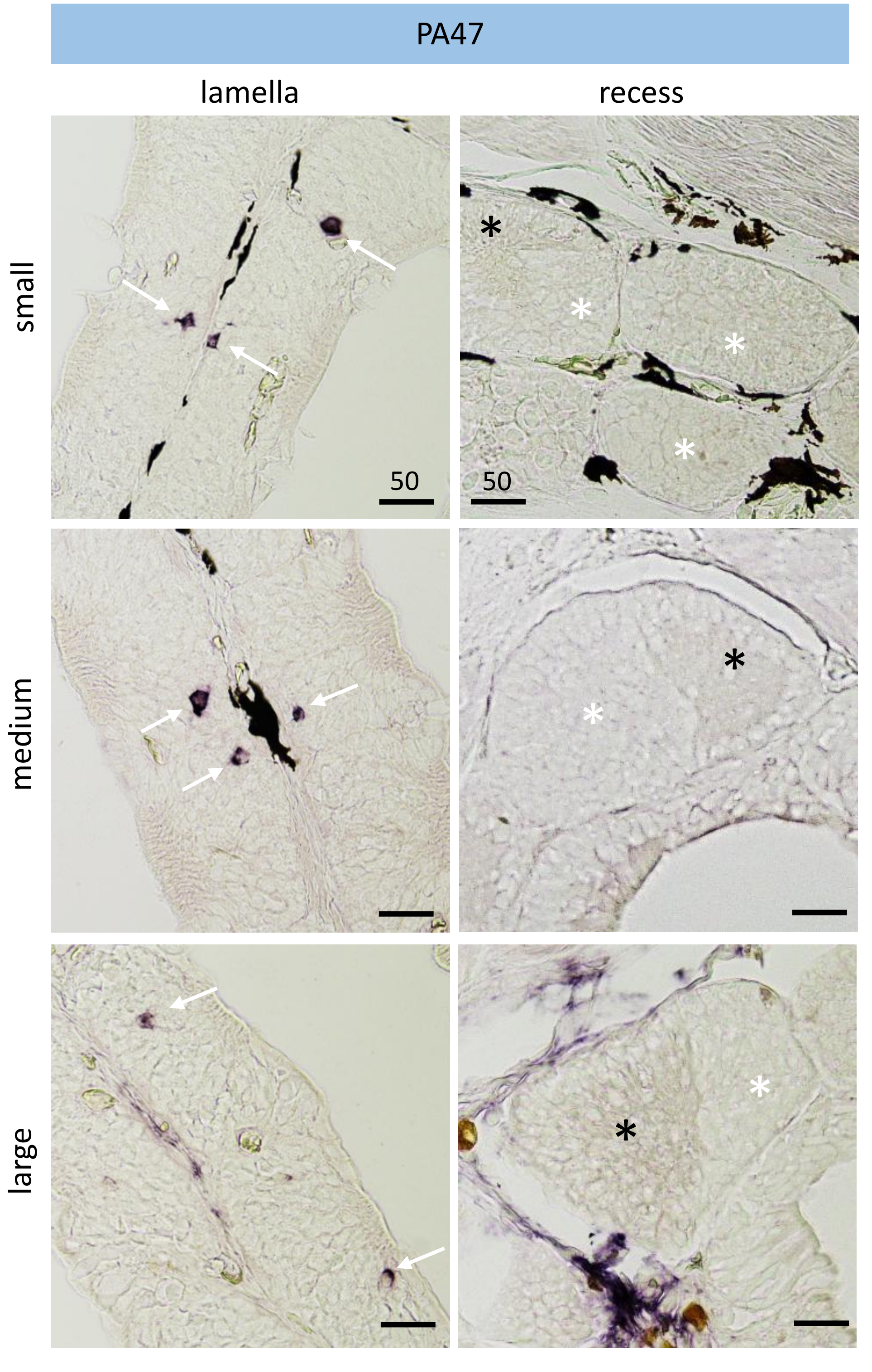


**FIGURE S2** Restricted expression of PA47 in the small, medium and large *Protopterus annectens*. In all specimens, PA47 is expressed in the lamellar OE (arrows) but not in the RecE (white asterisks). Black asterisks indicate the glandular epithelium in the recesses. Scale bars: 50 μm.

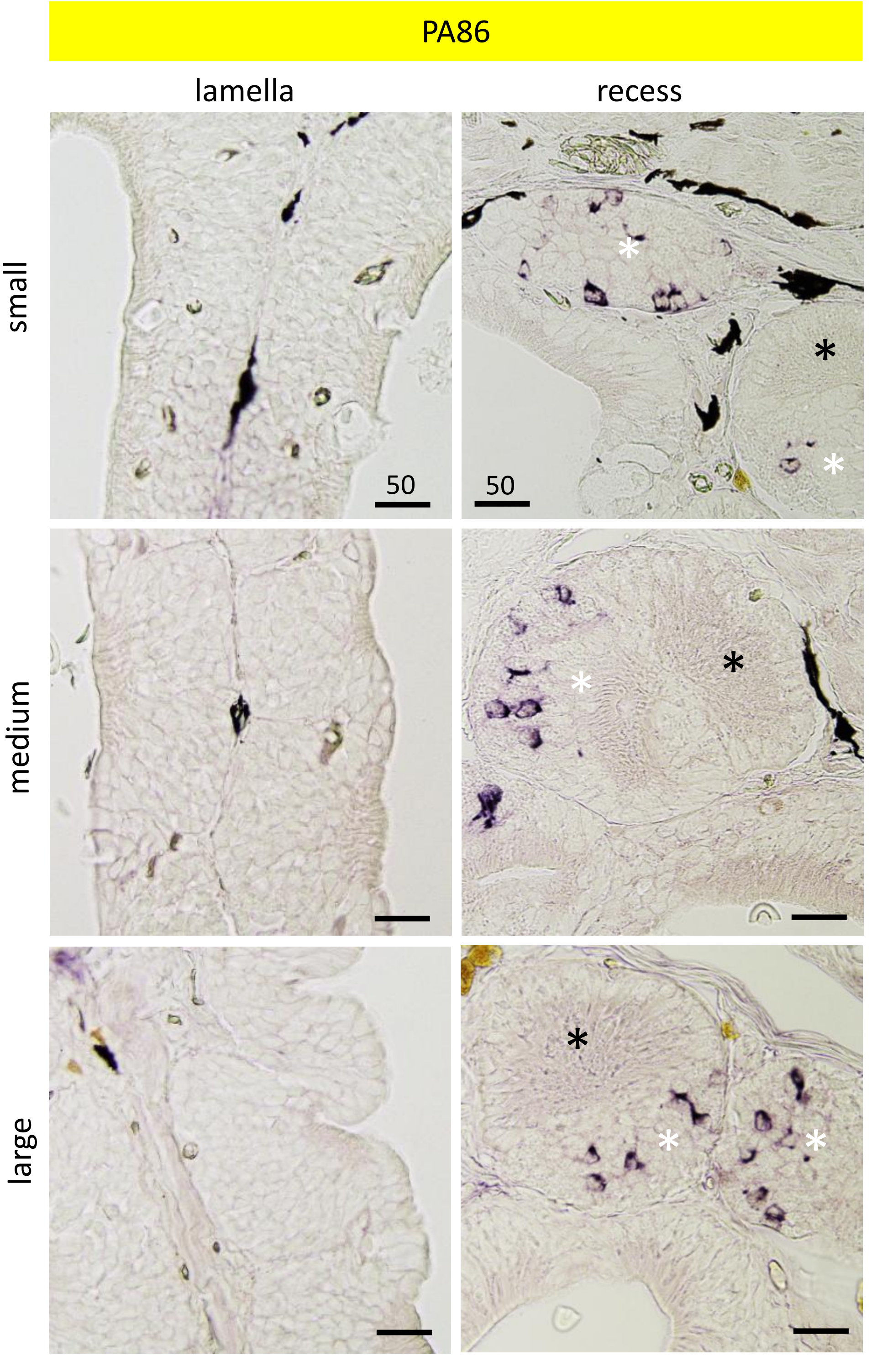


**FIGURE S3** Restricted expression of PA86 in the small, medium and large *Protopterus annectens*. In all specimens, PA86 is not expressed in the lamellar OE but is expressed in the RecE (white asterisks). Black asterisks indicate the glandular epithelium in the recesses. Scale bars: 50 μm.

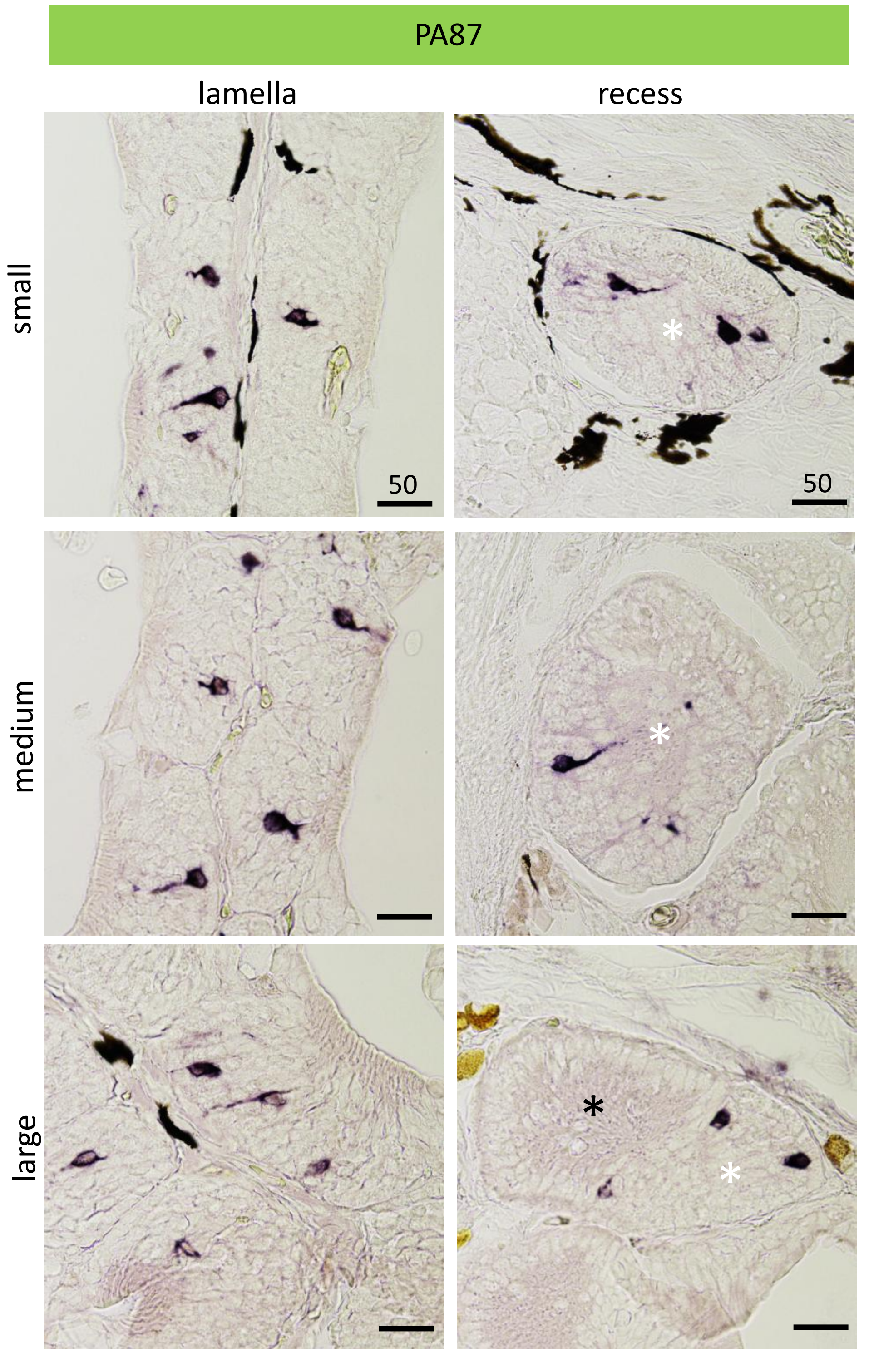


**FIGURE S4** Expression of PA87 in the small, medium and large *Protopterus annectens*. In all specimens, PA87 is expressed both in the lamellar OE and in the RecE (white asterisks). Black asterisks indicate the glandular epithelium in the recesses. Scale bars: 50 μm.
