# Supplementary material for: Shifts in type 2 vomeronasal receptor expression during postnatal development in the lungfish olfactory organ
Source: J Anat. 2026 Mar 8:10.1111/joa.70129. Online ahead of print. doi: 10.1111/joa.70129 (PMC13398680; doi:10.1111/joa.70129)
Supplement: Supplementary file 1 — Data S1. [file JOA-9999-0-s002.docx]

>annecV2R63

ATGATTGCAAATCAGACAGATGCTACAAACTGCATATCTTGTCCTGAAGAGTACTGGTCGGATGCTGCCCATGAGAAATGTGTGCCAAAAATCACTGAGTTCTTGTCATATGAAGAGCCTTTGGGCACGGCACTGACTGCCTTCGCTATATTATTTTCACTGGTTACTGTTTCAGTGATTGGAACATTCCTCCACTTCCGTGAAACACCAATAGTTAAAGCTAACAATCGTGAACTCAGTTACTTACTGCTTCTTTCGTTATTGATGTGCTTTTTGTGTTCCCTTCTATTTATTGGTCAACCTCAAACTCTGAGCTGCATGCTCCGCCAGGCAGCATTTGGAATTGTGTTCTCCTTATGTCTATCTTCTATCCTTGCAAAGACATTTATTGTTATAATTGCATTCAGAGCCACAAAGCCTGGTAGCAGTTTGAAGAGGTGGGTGGGATCCAGGGTACCATATTCAGTTGTGACCATCTGTTCTTCACTCCAGGTTTTGATTTGCATGTTTTGGGTGAGAACCTTTCCTCCTTTCAGTGAATGGAACAGGACAGTAGAAGAAGGAAAAATCCTTATTCAGTGTAATGAAGGGTCAGCAGTACGTTTCTGGTTCATATTGGGGTACCTTGGGATTTTGGGAAGCATCAGCCTCATCCTGGCATTCTTTGCCAGGAAACTTCCGGACAGCTTTAATGAAGCTAAGTTTATCACATTTAGTATGCTGGTTTTTGCCAGTGTGTGGCTGTCTTTTATCCCTGCGTACCTAAGCACAAAAGGAAAGGCCATGGTAGCTGTGGAAATATTTGCAATCCTGTGTTCTACAGCGGGTATACTTTTCTGTATATTTTCTCCAAAGTGTTATATTATTTTACTGAAGCCTGGAAGAAATACCCGTCACTATCTGATGGGAAAGGAAAACTCCATTTAA

>annecV2R114

GGAATATTGCTGATAGCTGCATCATTATTAGGAGCTTGTGTTACATGTGCAGTACTAATAGTCTTTGCATATTACAGAGATACACCTATTGTAAAAGGTAACAATTCAGAGTTAAGTTTCCTGATTTTGGTTTCTTTAGTACTCTGTTTTCTTTGTTCCCTAACATTTATTGGGGAACCATCACAGTGGTCTTGCATGCTGCGGAATACAATTTTTGGTATAACATTTGCACTTTGTTTTTCATGTATTCTAGCAAAAACAAGTGTTGTATTAATGGCTTTCAAAGCAACACTTCCAGGTAGTAAGGCAATCAAATGGTTCAAACCTGCTCAACAAAGAGCCTTAATATTTTTTGGAACAGCTATTCAGATTACAGTTTGCATTGCATGGCTGTTCATAGCACCCCCATTTCCCAATCAAAACACACAAGTGCAGACTTCGAAAATTATTCTTGAATGTGCTGTGGGCTCATTAACTTTCTTTTCATTTGTACTGGGTTACATTGGGTTCTTGTCATGTTTTAGTTTTGTGTTTGCTTTTCTAGCTCGTGAACTGCCAAGTAATTATAATGAGGCAAAACATATTACCTTCAGCATGGTCATTTTTCTAACTGTATGGATTGCCTTTATACCAGCGTATATTAGCACACATGGAAAATATACAATTGCTGTACAAATATTTGCAATTCTGACATCAACATTTGGTTTACTTATTTGTATATTTGCCCCAAAGGTTTATCTAATTTTACTAAAGCCAGAAGAAAATACTAAGAAAAGGCTGCTGAATAGAGTAGTAAGTGAATAG

>annecV2R120

ATGACTGATTCAAGTAATTGTATTAAGTGCCCAGAAGAGATGTGGCCCAACAAGGAACAAGAGAAGTGCATCCCTAAAGTAATCGACTTCCTCACCTTTGACACATTTTTGGGCTCCGTCTTAGCAACGCTGTCGATTTTCTTTACTTTACTAACAGTTGCTGTGCTTTTCACCTTCATTAGTTTTCGAGCCACTCCAATTGTTCGAGCTAACAATCGTGAGCTCAGTTACTTTCTTCTTATTTGTCTAATGATGTGCTCCCTCTGCTGCCTTATTTTCATTGGACGTCCTAAAAAGCTGAATTGTCTCCTTCGCCAGGCTGCATTTGGGATTATATTTTCGGTCTGCATTTCTGCTATAATGGCAAAAACCATGACTGTAGTTATTGCATTTAATGCAACAAATCCCTCCAGCAAGCTGTTGGTAGGTTCTAAATTGCCAAATTACTTTATTATCCTCAGCTCATCCATTCAAGTTATAATATGCATTACATGGCTGTGTATAGCCCATCCTTTCCCATATAACAATATGGATACTGAAGAAGGAAAAATAATAATTGAATGTAATGAAGGGTCTAATCTCATGTTTTGCATAATGCTTGGGTACATGGGTCTACTTGCTACTGTTAGTTTCATACTTGCTTTTCTTGCAAGGAACCTGCCTGATGCCTTCAATGAAGCCAGGTATATTACATTTAGTATGCTAGTTTTCTGTGCAGTCTGGCTTTCTTTTATACCTGCCTATATAAGTACAAGAGGCAAATATACAGTAGCAGTAGAGATATTTACTATTTTATGCTCTAGTGCAGGTCTGCTTGGATGCATATTTTTTCCAAAATGCTACATTATTTTACTGAGACCAGAGAAGAATACTAAGCAGAAGGTAATTGGGAAAGAAAAGGGGTCACATATCAGATGA

>annecV2R217

TGCAGGGCCTGTCCATCTGAAGAATGGTCGAATCTGCAAAAGCGCAAGTGTATTCAGAGATCTACAGAGTTCCTCTCCTATGACGATCCTTTGGGCATTGCATTAGCATCAGTCACTATTAGCTGTACAGCTCTGACACTGGTCATCTTTGCTACATTTATCAAGTTCAGGAAGACTCCAATTGTCAAGGCTAATAATCAAAACCTTAGCTTTCTTCTCCTTGTCAGCCTTTTCTTCTGCTTCCTGTGTTCCTTGATTTTTATTGGACTGCCTAACAGAGTTTCCTGCAAGCTTTGTCAAGCAGTGTTTGGTATAATCTTTGCTCTTTCTGTCTCCTGCATGTTGTCAAAGACAATTACTGTAGTCCTGGCCTTCAATGCTGCAAAACCTAACAGTCTGTTGAGAAGATGGGTTCGACCACAGACCTCTTACCTTATAATAGTAATGTGTACATTTTTTCAGGTTATCATATGTACCAGCTGGATAATCTTCTCATCTCCATTTCTAGAAAGAAACTCTAAAACCCAGCCAGGGCTTATTATTATTCAGTGCAATGAAGGTTCCACCATTGCTTTCTGGTGCATGCTGGGATACATAGGCCTCCTGGCAGTAATCTGTTTTGTTGTTGCATTTCTGTCTAGGAAACTGCCGGACAGCTTTAATGAGGCTAAGTTCGTCACATTTAGTATGGTTGTATTTCTGGGGGTTTGGCTACCTTTTATACCTGCTTACCTTAGTACTAAGGGAAAATACATGGTGGCAGTAGAGATATTTGCTATAATTGCTTCGAGTCTGGGTTTGCTTGTCTTAATATTTTTCCCAAAGTGTTACATCATTCTAACGCAACCTTACAGAAATACAAAGGAGTACATAATACGAAAAAAGAACACGGAGTGA

>annecV2R276

ATGGAACGTGATAAGTGTATCCCCAAAGTGATTGATTTTCTGAGTTACGAGGAGCCTCTTGGTGCAACTTTAGCAGCGGTTGCTGTTTTCTTATGCATTGGCACTGTGTGTACCTTGTGCATTTTTATTGCATACCGAGAAACTCCAATAGTTAAAGCCAACAATCGAGAACTCAGCTACATTCTTCTAATGGCTCTCATGCTCAGCTTCCTGTGCTCCTTACTGTTTCTTGGTCAACCAACCAAAGTGACATGCATGCTCCGTCAGACAATTTTTGGCATTATTTTTTCCATCAACATTTCATCTGTTCTTGCTAAAACCATTACTGCATTTATTGCCTTCAAGATAAAAACTCCAAAGAAGAAATCATGGAAGTGGACTGGATATAGAATGCCTTACTTAGTGGTGATATTATGTTCTTTTGTACAAGTCATGATATGTATTATTTGGCTAATAGTTGCTTCACCGTTTCCAGAATTAAATGCAATGTTAGATGAAAGCAAAGTAATTTTTGAGTGTAATGAAGGTAAAAGCATATTTTTCTATTGCATGATAGGATATTTAGGAATTTTAGCAACAATGGCACTTTCAGTTGCCTTTCTGGCAAGAAAATTACCCAGTAGTTTTAATGAGGCCAGGCATATCACCTTTAGCATGTTTGCTTTTCTCAGTCTTTGGCTTTCATTCATTCCAGCCTATCTAAGCGCAAAGGATAAATACCTGGTTGCTGTAGAGATATTTGCCATTTTGTCTTCCAGCACCAGTATTTTGACTTGTATTTTTCTTCCAAAATGTTATATGATAATGTTCAGGCCCCAGCCAGTAAAAAATAAGAAAGTTTTATTAACTAAAAAAAATAATGCAAACTAA

>annecV2R392

ATGTGGAGTGGAGGATACTTGCAAACTCCAGAGTCTGTCTGCAGTCAGAGTTGCCATATTGGGTATAGAAAAGCTCCACTTCTAGGGAGGCCTGTCTGCTGTTATGCCTGTGTGTTGTGCTCTGATGGAGAAATTTCAAATGAAACAGATGCTACAAACTGTCTGAAATGCTCAGAAGATACTTGGTCTGACGACGGAAGGACAGGCTGCATTTCAAAGCTTACAGAATATTTGTCTTTTGATGATCCTTTGGGTTCAACATTGACTGGTGTCATTGCTTTTTTTTCTGCTTTTCCAGCTCTCATATTGTGCATTTTTGTTACACATCGTGACACACCCATAGTGAAAGCAAACAACCGCAATCTGAGCTACTTTCTTCTAGTGGGTCTAGTTCTCTGCTTTCTCTGCTCCATTATATTTATTGGAAAACCTAACAAAATGAGCTGCATGGTCAGACAGACCTCATTTGGTATAACATTTTCTGTCTGCATTTCTGTTATTTTAGCAAAAACAATCACTGTTGTCATTGCTTTTCATGTAACAAGACCGGGAAGTTTGCAAAAAAGATGGGTGGGGTCCAACACTCCAAGCATGACTGGTCTTTTCTCTGCTGCTGTGCAGGTCATTATATGCATCATCTGGCTTGCTTATTCCGCACCTTTTCCAGAAAAAAACACAAATACTTTCAAGGAAAAAATAATTGTTGAATGCAATGAAGGATCTCTGACAATGTTCTACTCTATGCTGGGATACCTTGCATTACTGGCCACTATTTGCTTCATAGTTGCATTTTTAGTAAGGAAATTACCAGATAGGTATAATGAAGCTAAATTCATCACATTCAGTATGATCATTTTTGTGAGTGTTTGGCTGTCATTCATACCAGCATATCTGAGCACCAAAGGGAAATATGTAGTCGCT

>annecV2R467

TGTCAGAAGTGCTTAGACTATGACTGGTCCAATGAGAGGCAAGACAAGTGCATTCCAAAAGTCATTGAATTTCTGTCCTATGAAGAGCCACTTGGGTTCACTTTAGCTTCCATTACTGGTGTTTTTACCTTAATAAATGCAATGGTCTTTTGTATATTTCTGAAAAACAATGACACAGCCATTGTTCGGGCAAACAACAGGGACCTCAGCTACCTTCTCCTTTTGTCTCTCACGCTGTGCCTTTTGTGTGCTCTGTTGTTCATTGGACGCCCTTCCAGCTTGAGTTGTATCATCTCTCAGACTACCTTTGGGATTACTTTTTCTACGTGTGTCTCTTGTATTCTGGCAAAAACTATTACAGTTATACTTGCCTTCCGTTCTACACAACCTGGAAGTAACATAAGGAAATGGATGGGTCCAAGAACATACGGCTCTGTTGTAATTGGTGGCACAGCACTTCAGATGATAATATGTACTGTTTGTCTATCAGTTTCTTCAAGTTTTCTTGAAATTAGTATTGAACTTGACGATGAGAAGATAACTCTTCATTGTAATGAAAGTTCAAATGTCACATTTTGGTGCATGCTGGGATACCAGGCAGCACTTGCTGGCCTCTGTTTGCTACTTGCTTTCTGTGCAAGATCATTGCCAGATAGCTTCAATGAGGCAAAATTAATAACATTCAGTATGATAGTTTTCCTCACTGTATGGCTCTCATTTATTCCTGCCTATTTAAGTACAAAGGGAAAATACATGGTTGCTGTGGAGATATTTGCCATTCTGTCATCCAGTGCAGGGATGCTGGGTTGTATCTTC

>annecV2R488

TGCCTGTCTTGCCCATATGATCAGTGGCCCACAGAATCAAGGGATAAATGCATTGAGAAATCTGTAGAATTCCTCTCCTACAATGATCCACTAGGATTAGCTCTCGCCTTCATCTCCTCCTTCAGTGCTGTGCTACCTGCAGCTATCCTCATCATCTTCATCAAATACCATAACACTCCTGTTGTAAAGGCCAATAATCGTGATATAAGTTATATCTTGCTTGTTGGACTCATGCTTTGTTTTTTATGCTCGCTGGTATTCATTGGGCATCCCACTACAGGAACGTGCATGATTCGCCAAGCTGCTTTTGGTGTCATTTTTGTCCTTTGTGTTTCCTGTGTGTTAGCTAAAACTGTGATGGTGGTTATTGCATTCAATGCTACAAAACCAAATAGTAACTTAAAAAAGTGGGTTGGGCCAAAATTACCAAATTCTATAGTATTTGTGTGTACCATTGTCCAGATTATTATATGTTCTGTCTGGCTAGCTACCTCCCCACCTTTTCTTGAGTTAAACCTCAAATTACAAACAGGGATGATAATAGTTCAGTGTAATGAAGGTTCCCCCACTGCATTTTGGTGTATGTTAGGATATGTTGGCCTTTTGTCAAGTATCAGCTTTGCAGTTGCTTTCTTGTCTAGAAACCTCCCTGATAGTTTCAATGAAGCTAAATTTATTACTTTCAGCATGATTGTCTTTGTAAGTGTGTGGTTATCTTTTATCCCAGCATATCTAAGCACACAAGGCAAGTATATGGTAGCTGTTGAAATTTTTGCTATATTTTCATCTAGCGCTGGTTTGCTGGCATGTATATTCTTTCCAAAATGCTACATAATTTTACTGAGGCCCACTATGAACACAAAAGAATATTTAATGGGAAAAGGAATACAGAATAATAAATGTATATAA

>annecV2R526

TGTATTGAATGCCCACATGACCACTGGCCTAATGAGAAACGAGACAAGTGTCTTCCAAAAGTCATTGAATTTCTATCCTATGAAGAACCACTGGGCATTGCTTTGGCAGCCATTACAGTTCTGTTTGCCTCCATCACATCGTGTATTGCTTTCATCTTTGTGAAGTATCGTGACACTCCAATTGTTAGAGCCAATAATCGGGAACTCAGCTACCTTCTCCTGCTGTCCCTTCTCTTCTGTTTCATGTGCTCCTTGATATTCATTGGTCAGCCAATGAAGACAACATGCATGTTACGACAAACAGCCTTTGGCATCATATTTTCTCTCTGTATCTCTTGCATCACAGCAAAGACTGTCACTGTAGTTATTGCTTTCAATGCCACCAATCCCAACAGCAAACTGAGAAAATGGGTGGGGTCTAAGACACCAAGCATTATTGTCATATTCTGTACAATTGTTCAAGTTGTTCTGTGCATTGCTTGGTTAATCCTATCCCCACCATTCCCAGACAACAATATGACATCACAAGATGGCAAGATCATTATTGAATGTAATGAAGGGTCTATCATCACTTTTTATTGCATGCTAGGATATATGGGATTTCTAGCCACTGTGAGCTTTTTAGTAGCATTTCTAGCTAGAACACTCCCTGACAGTTTTAATGAGGCAAAATTTATCACTTTCAGCATGCTTGTGTTTGCTAGTGTCTGGATCTCCTTTATTCCTGCTTATTTAAGTACACGAGGAAAGTATATGGTTGCTGTTGAAATCTTTGCTATTTTGTCATCAAGTGTTGGACTACTAGGCTGCATTTTCTTTCCTAAATGCTACATTATTTTGCTGAAGCCAGAACTGAATACCAAAGAAAATTTAATAGGGAAGGCAAACTTTACCCACAAAAAATGA

>annecV2R535

ATGTCATGTTCAGAAGGAGAATTTGCTAACGAAACAGGTGCTGTTGATTGTTTCAAGTGCCAGGATGATCAGATGTCGAATGAAAGAAGAGATGAGTGTTTGCCAAAGCCCTTGGAATTTCTCTCATATGAAGACGATTTAGGAGTAATCCTGACAGCATCAACCAGTGCCTTGGCATTAATGCCTGTAGCAATATTAGTGACTTTTGTACATTACAGAGACACTCCTCTGGTCAAAGCCAACAACCGTGACATTAGCTATGTTCTGCTACTTGCTCTGACACTATGCTTTCTCTGTTCTTTTACCTTCATTGGTCGCCCTACTGTCATAATTTGCTTCATCCGTCAACCTGCTTTTGGTGTAATTTTTGCCCTTTGCATTGGCTGTGTGCTTGCCAAAACTGTTATGGTGGTTGTAGCATTCAGTGCCACAAAGCCTAACAGTAATCTTAAGCATCTTGTAGGTTCTAAACTACCAAGTTCCATAATCACTCTGTGTACTGTGGGTCAGGTTATCATATGTGTCATGTGGTTAGCACTTTCACCACCATACCCTGAAGCAGATCTGAAATTTCATACCTCAAAGATAATCATAAAATGTAATGAAGGCTCCAATATTGCATTCTGGTCCATGCTAGGATATATGGGCTTGCTGGCTACGGTTAGTTTCTCTGTTGCATTCTTATCAAGAAAATTACCAGATAATTTTAATGAGGCTCAGCATATTACGTTCAGCATGATTTTATTTGTAAGTGTCTGGCTTTCCTTCATCCCAGCCTATCTTAGTACACAAGGGAAGTATATGGTTGCTGTGGAAATATTTGGCATTCTGTCTTCAAGTGCTGGTCTCCTGACTTGCATATTTTTTCCCAAATGTTACATAATTCTGTTAAAATCAGAGCTAAACACCAAGGAATTCCTAATGGGAAAAGAAACATCTAACAAAAGGAAACACAAATAA

>annecV2R709

ATGAGCAGCATTCTCTGGAATGAAGCATTTCCCCAGACTCCTACCTCTGTCTGCAGTGAGAAGTGCCTTCCTGGATATAGAAAAGCTGTTCGACTGGGGCAACCAATATGCTGCTTTGACTGCATCCCTTGCTCTGAAGGAAGTATATCTAATGAAAGCGATTCTCTGGATTGTGTTAAGTGCCCAGAAGATCAGTGGACTGATATACATCAGGAAAAATGTATATATAAAACACTAGAGTTCCTGTCTTATGATGACATCTTAGGTGCAGTTTTAGCTACCGTGTCCCTGTGTGCAACTCTTTTTACTACTGCAATCCTATTTATTTTTATTAGGCATCATAAAACACCAATTGTCAAAGCCAACAACAGTGAATTAAGTTACTTTCTCCTTGTATCTCTCTTACTGTGTTTCCTCTGCCCACTGATTTTTATTGGTTATCCTAATAACTTAACATGCATTTTTAGACAGATGGCATTTGGGTTAATATTTGCCCTTTGTGTTTCCTGTGTTCTAGCAAAAACAGTTATGGTTGTCATCGCATTTAATGCAACTAAACCAAACAGCAGCTTAAGAAAACTTGTTGGTTCTAGATTGCCATATACTATAATAATTGTATGTACCTCTTTGCAGCTGATTGTGTGCATAATTTGGCTAACTGTGCGTCCTCCATTTTCTGAGGAAAATTTTAGAACTCGTACTGGAAAGATCATTGTTGAATGCAATGATGGTTCAACAACTGCTTTCTGGTGTATGTTAGGGTACATGGGAGTCTTAGCTAGTGTAAGTTTTCTTGTAGCATTCTTGTCTAGGAACTTGCCAGACAGCTTCAATGAAGCCAGATACATCACATTTAGCATGATTGTATTTGTCAGTGTGTGGTTATCATTTATCCCAGCATATGTTAGTACAACAGGAAAATATATGGTTGCAGTTGAAATATTCGCCATAGTGTCATCTGGTATTGGCTTGCTTTCTTGTATTTTTTTTCCCAAATGCTATATTATTCTACTAAGACCACACTTGAATACTAGGGAACATCTTATGGGTAGAGGCATTCAAAAAAACTGA

>annecV2R755

GTTTTGACTTCTGCTACTGGAGTTTGTTCTACACTTGCTGCATTTGTTCTTTATATCTTTGTGAAGAACAAAGAGACACCTATAGTCAAGGCTAATAATCGTGAGCTGAGTTATGTATTGCTTTTTGCCCTTTTGCTTGGATCCCTGTCTCCATTACTGTTCATTGGACGTCCAACATCAATCACATGTATGCTACAGCAAACATTATTTGGCATTGCATTTTCCATTTGCGTGTCTTCAATCTTGGCCAAGACTGTTATGGTAGTAATAGCATTCCGAGCCACAGATCCCACTAGTGGACTAAAAAAATATTTAGGTTCAGCTGTACCAAATCTAATAGTTATTTTCTGCTCAGGCTTACAAGGAATAATATGTATGGTGTGGACAGCCACATTTCCTCCATTTCTGCAGTTCACCACAAGTTCAGAGACTGCTAAAGTTATCATTGAATGTAAGGAAGGCTCTGTTGTATTTTTTTATGTGATGCTAGCCTACATGGGTCTTCTGGCTTGTGTGAGCTTTGCAGTGGCCTTTGCTGCACGTAAATTGCCAGATATTTTTAATGAAGCAAAGCACATTACATTCAGTATGCTAGTGTTTGCTAGTGTGTGGCTAGCTTTTATCCCAGCATATCTGAGCACCCAAGGG

>annecV2R183

ATGAGTTTATCTCTCAAGGATGCCCAAATTGCATGGAATGGAGGTCAAAAACAGCCTCCTAGGTCTGTCTGCAGTGAAAGTTGTCTTACAGGCTTTAGAAAGGCACCACAGAGAGACAAATCTGCCTGTTGCTATGACTGCATTCCTTGTGCTGAAGGTGAAATTTCCAACTACAGTGATTCCACTGATTGTTTGGCCTGTCCATTTGAGGAGTGGCCCAATGAAAGAAGAGACAAGTGTGTTCCAAGATCGATAGAATTCCTTTCTTATAACGATGTCCTGGGTTCAGTTTTGGCCACCGTTGCTCTTATGAGTTCTTGTCTGCCAGTTGCTATTGTTTCTGTGTTTGTCAAGTTCAGCAACACACCCCTTGTCAGAGCTAACAATCGAAACATCAGTTATGTTCTCCTGGTAGCCCTTCTGCTGTGCTCCCTGTGTTCATTGCTTTTTATTGGACAGCCTAATAAAATGACCTGCAAACTTCGGCAGGTAGTGTTTGGAGTAACTTTTGCCCTTTCTGTTTCTTGTGTGCTGGCTAAGACCATTATGGTTGTGGTAGCCTTTAACGCAACAAAACCCAATAGTTCTTTTAGACAGTGGATTGGCCCACAGCTTTCTTACTCTATTGTATTTTTATGTACAGTTGTTCAGGTAATTATTTGTATGAACTGGATCATTCTTACATCTCCATTTCCTGAATGGAATTTTAAATCACAACCTGGACTGATAATAATTGAATGCAATGAAGGTTCTGCTGTTGCATTCTGGTGCATGCTGGGGTACATGGGATTACTGGGTGCCACATGTTTTACTGTAGCGTTTTTGTCCAGAAACCTTCCAGATAGCTTTAATGAGGCAAAATTCATTACATTCAGTATGGCTGTGTTTGTGAGTGTGTGGGCAGCTTTTATTCCAGCTTACCTCAGCACTAAGGGGAAATACATGGTAGCAGCAGAAATATTTGCTATTATTGCTTCTAGCCAGGGTTTGCTTCTTTGTATATTTTTGCCTAAATGCTATATAATTCTTGTGAGGCCGGACATTAATACA

>annecV2R201

ATGTTTGGTTCTCTGCAAGCAGCTGGTGTTCTTATTATCTTCATCAGGTTCAGAAGAACACCTCTTGTCAAAGCCAACAACCAGAACCTCAGCTTCCTGTTACTGGTGTCACTTCTGTTTTGCTTCTTGTGTTCATTGTTTTTTATTGGCCACCCTAACAAAGTGACCTGCAGATTGCGACAGGCTGTGTTTGGCATAACTTTTGTTCTTTCCTTGTCTTGTGTGCTTGCCAAGACAATTATTGTAGTTATTGCATTCAGAGCAACAAAGCCTAATACTCACATGAAGCAATGGATGGGTCCCCAGCTCACTTACATGACAGTAACACTGTGCACGTTTGTGCAGGTTATTATCTGCATGTCCTGGCTTGCATTTTTTTCTCCATACCCAGAAAAAAACTATAGTTTACAGCCAGGACAGATATTAATTGAATGCAATGAAGGCTCCACTATGGCATTCTGGTGCATGCTGGGATACATGGGGCTGCTGGGTAATATATGTTTTGTAGTGGCATTCCTGGCTAGAAATCTGCCGGATCGCTTTAATGAGGCCAGATACATCACTTTCAGCATGGTTATATTTGTGTCTGTGTGGTTTGCCTTTATCCCAGCCTACCTTGGCACAAAGGGGAAATACATGGTAGCTGTAGAGATATTTGCTATTATCATGTCTTCTCTAGGTTTACTTGTGTGCATATTTTTTCCAAAGGGCTATATAATT

>annecV2R202

ATGGTGTTCCTTTCCTATGAAGAGCCTCTAGGAGCTGCACTGGTCGTTATCACAATGTTCGGGTCTCTGCAAGCAGCTGGTGTTCTTATTATTTTTATCAGGTTCAGAAAAACACCTCTTGTCAAAGCCAACAACCGGAACCTCAGCTTCCTGCTACTGGTGTCACTTCTGTTCTGCTTCCTGTGTTCATTGTTTTTTATTGGCCACCCTAACAAAGTGACCTGCAGATTGCGACAAGCTGTGTTTGGCATAACGTTTGCTCTTTCCTTGTCTTGTGTGTTGGCCAAGACCATTACTGTAGTTATTGCATTCAGTGCAACAAAACCTAATGCCCACAGGAAACGATGGATGGGTCCCCAGCTCACTTACATGACAGTAACACTGTGCACGTTTGTGCAGGTTATTATCTGCATGTCCTGGCTTGCATTTTTTTCTCCATACCCAGAAAAAAACTATAGTTTACAGCCAGGACAGATATTAATTGAATGCAATGAAGGCTCCACTATGGCATTCTGGTGCATGCTGGGATACATGGGGCTGCTGGGTAATATATGTTTTGTAGTGGCATTCCTGGCTAGAAATCTGCCGGATCGCTTTAATGAGGCCAGATACATTACTTTTAGCATGGTTATATTTGTGTCTGTGTGGCTTGCCTTCATCCCAGCCTACCTTAGCACAAAGGGGAAATACATGGTAGCTGTAGAGATATTTGCTATTATCATGTCTACTCTAGGTTTACTTGTGTGCATATTTTTTCTGAAGGGCTATATAATTATACTGAGG

>annecV2R205

ATGGTGTTCCTTTCCTATGAAGAGCCTCTAGGAGCTGCACTGGTCGTTATCACAATGTTCGGGTCTCTGCAAGCAGCTGGTGTTCTTATTATTTTTATCAGGTTCAGAAAAACACCTCTTGTCAAAGCCAACAACCGGAACCTCAGCTTCCTGCTACTGGTGTCACTTCTGTTCTGCTTCCTGTGTTCATTGTTTTTTATTGGCCACCCTAACAAAGTGACCTGCAGATTGCGACAAGCTGTGTTTGGCATAACGTTTGCTCTTTCCTTGTCTTGTGTGTTGGCCAAGACCATTACTGTAGTTATTGCATTCAGTGCAACAAAACCTAATGCCCACAGGAAACGATGGATGGGTCCCCAGCTCACTTACATGACAGTAACACTGTGCACACTTGTACAGGTTATCATCTGCATAACATGGATTACAGTTTCTTCACCACACCCAGAAAACAACTATAATTTACAGCCAGGACAGATAGTAATTGAATGTAACGAAGGGTCCACTGTGGCATTTTGGTGCATGCTGGGATACATGGGGCTGCTGGGTAATATATGTTTTGTAGTAGCATTCCTGGCTAGAAATCTGCCAGATCGCTTTAATGAAGCCAGATACATTACTTTCAGCATGGTTATATTTGTGTCTGTGTGGTTTGCCTTTATCCCAGCCTACCTCAGCACAAAGGGGAAATACATGGTAGCTGTAGAGATTTTTGCTATTATCATGTCTACACTAGGTTTACTTGTGTGTATATTTTTTCCAAAGGGCTATATAATTATACTGAGACCTGATATTAACACAAAATATTATGTCTGTATTAATGAGCAAACACTGAAAAGAAGAGTGTAA

>annecV2R206

ATGGTGTTCCTTTCCTATGAAGAGCCTCTAGGAGCTGCACTGGTCGTTATCACAATGTTCGGGTCTCTGCAAGCAGCTGGTGTTCTTATTATTTTTATCAGGTTCAGAAAAACACCTCTTGTCAAAGCCAACAACCGGAACCTCAGCTTCCTGCTACTGGTGTCACTTCTGTTCTGCTTCCTGTGTTCATTGTTTTTTATTGGCCACCCTAACAAAGTGACCTGCAGATTGCGACAAGCTGTGTTTGGCATAACGTTTGCTCTTTCCTTGTCTTGTGTGTTGGCCAAGACCATTACTGTAGTTATTGCATTCAGTGCAACAAAACCTAATGCCCACAGGAAACGATGGATGGGTCCCCAGCTCACTTACATGACAGTAACACTGTGCACGTTTGTGCAGGTTATTATCTGCATGTCCTGGCTTGCATTTTTTTCTCCATACCCAGAAAAAAACTATAGTTTACAGCCAGGACAGATATTAATTGAATGCAATGAAGGATCCACTGTGGCTTTTTGGTGCATGCTGGGATACATGGGGCTGCTGGGTAATATATGTTTTGTAGTGGCATTCCTGGCTAGAAATCTGCCAGATCGCTTTAATGAAGCCAGATACATTACTTTCAGCATGGTTATATTTGTGTCTGTGTGGTTTGCCTTTATCCCAGCCTACCTCAGCACAAAGGGGAAATACATGGTAGCTGTAGAGATTTTTGCTATTATCATGTCTACACTAGGTTTACTTGTGTGCATATTTTTTCCAAAGGGCTATATAATTATACTGAGACCTGATATTAACACAAAATATTATGTCTGTATTAATGAGCAAACACTGAAAAGAAGAGTGTAA

>annecV2R208

ATGGTGTTCCTTTCCTATGAAGAGCCTCTAGGAGCTGCACTGGTCGTTATCACAATGTTCGGGTCTCTGCAAGCAGCTGCTGTTCTAATTATCTTTATCAGGTTCAGAGGAACACCTCTTGTCAAAGCCAACAACCAGAACCTCAGCTTCCTGCTACTGGTGTCACTTATGTTCTGCTTCCTGTGTTCTTTGCTTTTTATTGGCCGTCCTAACAAAGTGACCTGCAGATTGCGACAAGCTGTGTTTGGCATAACGTTTGCTCTTTCCTTGTCTTGTGTGTTGGCCAAGACCATTACTGTAGTTATTGCATTCAGTGCAACAAAACCTAATGCCCACAGGAAACGATGGATGGGTCCCCAGCTCACTTACATGACAGTAACACTGTGCACGTTTGTGCAGGTTATTATCTGCATGTCCTGGCTTGCATTTTTTTCTCCATACCCAGAAAAAAACTATAGTTTACAGCCAGGACAGATATTAATTGAATGCAATGAAGGATCCACTGTGGCTTTTTGGTGCATGCTGGGATACATGGGGCTGCTGGGTAATATATGTTTTGTAGTGGCATTCCTGGCTAGAAATCTGCCAGATCGCTTTAATGAAGCCAGATACATTACTTTCAGCATGGTTATATTTGTGTCTGTGTGGTTTGCCTTTATCCCAGCCTACCTCAGCACAAAGGGGAAATACATGGTAGCTGTAGAGATTTTTGCTATTATCATGTCTACACTAGGTTTACTTGTGTGCATATTTTTTCCAAAGGGCTATATAATTATACTGAGACCTGATATTAACACAAAATATTATGTCTGTATTAATGAGCAAACACTGAAAAGAAGAGTGTAA

>annecV2R211

ATGTTTGGTTCTCTGCAAGCAGCTGGTGTTCTTATTATCTTCATCAGGTTCAGAAGAACACCTCTTGTCAAAGCCAACAACCAGAACCTCAGCTTCCTGTTACTGGTGTCACTTCTGTTTTGCTTCTTGTGTTCATTGTTTTTTATTGGCCACCCTAACAAAGTGACCTGCAGATTGCGACAGGCTGTGTTTGGCATAACTTTTGTTCTTTCCTTGTCTTGTGTGCTTGCCAAGACAATTATTGTAGTTATTGCATTCAGAGCAACAAAGCCTAATACTCACATGAAGCAATGGATGGGTCCCCAGCTCACTTACATGACAGTAACACTGTGCACGTTTGTGCAGGTTATTATCTGCATGTCCTGGCTTGCATTTTTTTCTCCATACCCAGAAAAAAACTATAGTTTACAGCCAGGACAGATATTAATTGAATGCAATGAAGGCTCCACTATGGCATTCTGGTGCATGCTGGGATACATGGGGCTGCTGGGTAATATATGTTTTGTAGTGGCATTCCTGGCTAGAAATCTGCCGGATCGCTTTAATGAGGCCAGATACATTACTTTTAGCATGGTTATATTTGTCTCTGTGTGGTTTGCCTTCATCCCAGCCTACCTCAGCACAAAAGGTAAATACATGGTAGCTGTAGAGATATTTGCTATTATCATGTCTTCTCTAGGTTTACTTGTGTGCATATTTTTTCCAAAGGGCTATATAATTATACTGAGACCTGATATTAACACAAAATATTATGTCTGTATTAATGAGCAAACACTGAAAAGAAGAGTGTAA

>annecV2R213

ATGGTGTTCCTTTCCTATGAAGAGCCTCTAGGAGCTGCACTGGTCGTTATCACAATGTTCGGGTCTCTGCAAGCAGCTGGTGTTCTTATTATTTTTATCAGGTTCAGAAAAACACCTCTTGTCAAAGCCAACAACCGGAACCTCAGCTTCCTGCTACTGGTGTCACTTCTGTTCTGCTTCCTGTGTTCATTGTTTTTTATTGGCCATCCTAACAAAGTGACCTGCAGATTGCGACAAGCTGTGTTTGGCATAACGTTTGCTCTTTCCTTGTCTTGTGTATTGGCCAAGACCATTACTGTAGTTATTGCATTTAGTGCAACAAAACCTAATACCCACAGGAAACGATGGATGGGTCCCCAGCTCACTTACATGACAGTAACACTGTGCACGTTTGTGCAGGTTATTATCTGCATGTCCTGGCTTGCATTTTTTTCTCCATACCCAGAAAAAAACTATAGTTTACAGCCAGGACAGATATTAATTGAATGCAATGAAGGCTCCACTATGGCATTCTGGTGCATGCTGGGATACATGGGGCTGCTGGGTAATATATGTTTTGTAGTGGCATTCCTGGCTAGAAATCTGCCGGATCGCTTTAATGAGGCCAGATACATTACTTTTAGCATGGTTATATTTGTCTCTGTGTGGTTTGCCTTCATCCCAGCCTACCTCAGCACAAAAGGTAAATACATGGTAGCTGTAGAGATATTTGCTATTATCATGTCTTCTCTAGGTTTACTTGTGTGCATATTTTTTCCAAAGGGCTATATAATTATATTGACGCCTGATATTAATACAAAACATTATTTATGCAACAATGAGCAAATACTGAAAAGAAGATAG

>annecV2R214

ATGGTGTTCCTTTCCTATGAAGAGCCTCTAGGAGCTGCACTGGTCGTTATCACAATGTTCGGGTCTCTGCAAGCAGCTGCTGTTCTAATTATCTTTATCAGGTTCAGAGGAACACCTCTTGTCAAAGCCAACAACCAGAACCTCAGCTTCCTGCTACTGGTGTCACTTATGTTCTGCTTCCTGTGTTCTTTGCTTTTTATTGGCCGTCCTAACAAAGTGACCTGCAGATTGCGACAAGCTGTGTTTGGCATAACGTTTGCTCTTTCCTTGTCTTGTGTGTTGGCCAAGACCATTACTGTAGTTATTGCATTTAGTGCAACAAAACCTAATACCCACAGGAAACGATGGATGGGTCCCCAGCTCACTTACATGACAGTAACACTGTGCACGTTTGTGCAGGTTATTATCTGCATGTCCTGGCTTGCATTTTTTTCTCCATACCCAGAAAAAAACTATAGTTTACAGCCAGGACAGATATTAATTGAATGCAATGAAGGCTCCACTATGGCATTCTGGTGCATGCTGGGATACATGGGGCTGCTGGGTAATATATGTTTTGTAGTGGCATTCCTGGCTAGAAATCTGCCGGATCGCTTTAATGAGGCCAGATACATTACTTTTAGCATGGTTATATTTGTCTCTGTGTGGTTTGCCTTCATCCCAGCCTACCTCAGCACAAAAGGTAAATACATGGTAGCTGTAGAGATATTTGCTATTATCATGTCTTCTCTAGGTTTACTTGTGTGCATATTTTTTCCAAAGGGCTATATAATTATATTGACGCCTGATATTAATACAAAACATTATTTATGCAACAATGAGCAAATACTGAAAAGAAGATAG

>annecV2R244

ATGGTCTGTCCATCTGAAGAATGGCCTAATGAAAGAAAAGACAAGTGCTTCCCAAGTTCAGCAGAATTCCTTTCTTACAGTGATATCCTGGGTGCTAGTTTGGCTATGATAGGAGTTACCAGTTCCCTGCTGCCAGTTGCCATATTTATAATCTTTGTTAGATATGGTGACACACCTCTTGTCAGAGCCAACAATCGAAACATCAGTTACCTGCTCCTGGTATCCCTTCTTCTGTGTTTCCTGTGTTCCTTGCTTTTCATTGGACATCCTTCTACAGTGACCTGCAGACTTCGTCAGGTGGTGTTTGGTATAACCTTTGCCCTGTCTGTCTCCTGTGTTTTGGCTAAAACCATTATGGTTGTGGCAGCCTTCAATGCTACAAAACCCAACAGCCACTTAAGAGGTTGGGTCAGACCACAAACTTCTTACAGCATTGTATTTATATGTACACTTGTTCAGGTAGTTATTTGTATGAACTGGATTATTTTTGCCTCTCCATATCCTGAAAGGAATTTTAATTCACAGCCTGGGGTGGTAATAATTGAATGTAACGAAGGTTCTACTATCACATTCTGGTGCATGCTGGGATATATGGGATTACTTGCTGGTACCTGTTTTGTTGTAGCATTTCTATCTAGAAACCTTCCAGACAGCTTTAATGAGGCAAAGTTCATCACATTCAGCATGGTTGTGTTTGCCAGTGTGTGGATGGCCTTTATCCCAGCATACCTCAGCACTAAGGGTAAATATATGGTAGCAGTGGAAGTATTTGCTATTATTGCTTCTAGCCTGAGTTTACTTGTTTTTATATTTTTTCCTAAGTGCTATATCATTCTAATTAGACCAGACATTAATACCAAAGAATATTTAAGGGGAAAAGTAATGAAGTGA

>annecV2R352

ATGTGGAACAGTGGTCTCAATAAGTCTCCCAGGTCCGTCTGTAGTGAAAGTTGCCCTCCAGGATTCAGAAAGGTTGCACAAACAGGGAAGCCTGTATGCTGCTATGTTTGTGCTCCCTGCGCTCAAGGAGAAATTGCTAATGAGAGTGATTCCAAAGCTTGTACAGCGTGTCCACCAGAAGAATGGCCCAATGAAAGAGGGTATAAGTGCATCCGGAGGTCAACAGAATTCCTATCATATGAGGATCCCTTCGCTGTTGCTTTCATCTTCATTGCAATTTTCTTCTCATTACTGACACTTTCTGTGTTTGTCATATTTGTCAGATTTAATGACACGCCTGTTGTAAAAGCCAATAATCAAAATATGAGTTATCTACTCCTTGGCTCCCTCTTCCTCTGTTTTCTGTGTTCTTTGGTATTTATTGGACATCCTAACAAAGTTACCTGTAAATTTAGGCAAGTAATATTTGGTGTAACCTTTGTTCTTTCTGTTGCTTGCATCTTAGCTAAGACTATATTGGTTGTTGTAGCTTTCAATGCAAACAAACCCAACAGCTTCTTAAGAAGATGGGTGGGTCCACAGCTGCCTTATGCCATAGTATTACTCTGCACATTTGTTCAGCTTATTATATGTGCTGTATGGGTTAGCTTCTATTCTCCTTTTCCAGAACAGAATTTTAAGGCAAAAGCAGGGATGATAATAATTGAATGTAATGAAGGTTCACCAGTTGCATTCTGGTGCATGCTGGGATATATGGGACTATTGGCTGCTGTCTGCTTTGCTGTTGCATATCTGTCTAGACACCTACCAGATAGTTTTAATGAGGCTACATTCATTGCCTTCAGCATGACTATTTTTGTTACTGTTTGGTTGGCCTTTATCCCAGCATACCTCAGTACAAAGGGCAAATACATGGTCGCAGTAGAGATATTTGCTATTATTGTTTCTAGTGCAGGTCTGCTTACAAGCATATTTTTACCAAAGTGCTACATCATTCTAATAAGGCCTGATATGAACAAAAAAGAATATTTAATGGGAAAGGGAACAAAACATGATTTAGCAACATAG

>annecV2R353

TGTACAGCGTGTCCACCAGAAGAATGGCCCAATGAAAGAGGGTATAAGTGCATCCGGAGGTCAACAGAATTCCTATCATATGAGGATCCCTTCGCTGTTGCTTTCATCTTCATTGCAATTTTCTTCTCATTACTGACACTTGCTGTCTTTGTCATATTTGTCAGATTTAATGACACGCCTGTTGTAAAAGCCAATAATCAAAATATGAGTTATCTACTCCTTGGCTCCCTCTTCCTCTGTTTTCTGTGTTCTTTGGTATTTATTGGACATCCTAACAAAGTTACCTGTAAATTTAGGCAAGTAATATTTGGTGTAACCTTTGTTCTTTCTGTTGCTTGCATCTTAGCTAAGACTATATTGGTTGTTGTAGCTTTCAATGCAAACAAACCCAACAGCTTCTTAAGAAGATGGGTGGGTCCACAGCTGCCTTATGCCATAGTATTACTCTGCACATTTGTTCAGCTTATTATATGTGCTGTATGGGTTAGCTTCTATTCTCCTTTTCCAGAACAGAATTTTAAGGCAAAAGCAGGGATGATAATAATTGAATGTAATGAAGGTTCACCAGTTGCATTCTGGTGCATGCTGGGATATATGGGACTATTGGCTGCTGTCTGCTTTGCTGTTGCATATCTGTCTAGACACCTACCAGATAGTTTTAATGAGGCTACATTCATTGCCTTCAGCATGACTATTTTTGTTACTGTTTGGTTGGCCTTTATCCCAGCATACCTCAGTACTAAGGGCAAATACATGGTAGCAGTAGAGATATTTGCTATTATTGTTTCTAGTGCAGGTCTGCTTACAAGCATATTTTTCCCAAAGTGCTACATCATTCTAATAAGGTCTGACATGAACAAAAAAGAATATTTAATGGGAAAGGGAGCAAAACATGATTTAACAACATAG

>annecV2R354

TGTACAGCGTGTCCACCAGAAGAATGGCCCAATGAAAGGAGGTATAAGTGCATCCGGAGGTCAACAGAATTCCTATCATATGAGGATCCCTTCGCTGTTGCTTTCATCTTCATTGCAATTTTCTTCTCATTACTGACACTTGCTGTCTTTGTCATATTTGTCAGATTTAATGACACGCCTGTTGTAAAAGCCAATAATCAAAATATGAGTTATCTACTCCTTGGCTCCCTCTTCCTCTGTTTTCTGTGTTCTTTGGTATTTATTGGACATCCTAACAAAGTTACCTGTAAATTTAGGCAAGTAATATTTGGTGTAACCTTTGTTCTTTCTGTTGCTTGCATCTTAGCTAAGACTATATTGGTTGTTGTAGCTTTCAATGCAAACAAACCCAACAGCTTCTTAAGAAGATGGGTGGGTCCACAGCTGCCTTATGCCATAGTATTACTCTGCACATTTGTTCAGCTTATTATATGTGCTGTATGGGTTAGCTTCTATTCTCCTTTTCCAGAACAGAATTTTAAGGCAAAAGCAGGGATGATAATAATTGAATGTAATGAAGGTTCACCAGTTGCATTCTGGTGCATGCTGGGATATATGGGACTATTGGCTGCTGTCTGCTTTGCTGTTGCATATCTGTCTAGACACCTACCAGATAGTTTTAATGAGGCTACATTCATTGCCTTCAGCATGACTATTTTTGTTACTGTTTGGTTGGCCTTTATCCCAGCATACCTCAGTACTAAGGGCAAATACATGGTAGCAGTAGAGATATTTGCTATTATTGTTTCTAGTGCAGGTCTGCTTACAAGCATATTTTTCCCAAAGTGCTACATCATTCTAATAAGGTCTGACATGAACAAAAAAGAATATTTAATGGGAAAGGGAGCAAAACATGATTTAACAACATAG

>annecV2R350

TGTACAGCGTGTCCACCAGAAGAATGGCCCAATGAAAGGAGGTATAAGTGCATCCGGAGGTCAACAGAATTCCTATCATATGAGGATCCCTTCGCTGTTGCTTTCATCTTCATTGCAATTTTCTTCTCATTACTGACACTTGCTGTCTTTGTCATATTTGTCAGATTTAATGACACGCCTGTTGTAAAAGCCAATAATCAAAATATGAGTTATCTACTCCTTGGCTCCCTCTTCCTCTGTTTTCTGTGTTCTTTGGTATTTATTGGACATCCTAACAAAGTTACCTGTAAATTTAGGCAAGTAATATTTGGTGTAACCTTTGTTCTTTCTGTTGCTTGCATCTTAGCTAAGACTATATTGGTTGTTGTAGCTTTCAATGCAAACAAACCCAGCAGCTTCTTAAGAAGATGGGTGGGTCCACAGCTGCCTTATGCCATAGTATTACTCTGCACATTTGTTCAGCTTATTATATGTGCTGTATGGGTTAGCTTCTATTCTCCTTTTCCAGAACAGAATTTTAAGGCAAAAGCAGGGATGATAATAATTGAATGTAATGAAGGTTCACCAGTTGCATTCTGGTGCATGCTGGGATATATGGGACTATTGGCTGCTGTCTGCTTTGCTGTTGCATATCTGTCTAGACACCTACCAGATAGTTTTAATGAGGCTACATTCATTGCCTTCAGCATGACTATTTTTGTTACTGTTTGGTTGGCCTTTATCCCAGCATACCTCAGTACAAAGGGCAAATACATGGTCGCAGTAGAGATATTTGCTATTATTGTTTCTAGTGCAGGTCTGCTTACAAGCATATTTTTACCAAAGTGCTACATCATTCTAATAAGGCCTGATATGAACAAAAAAGAATATTTAATGGGAAAGGGAACAAAACATGATTTAGCAACATAG

>annecV2R357

ATGTGGAACAATGGTTATACACAGCCTCCCAGATCAGTCTGCAGTGAAAGCTGCCTCCCAGGGTTTAGAAAATCTGCCCAGAATGGGAAACCTGAATGCTGTTATGACTGTGTACCATGTTCTGAAGGAGAAATATCCAACCAGAGTGATTCCAATATCTGCCTGGTATGTCTTCCAGAAGATTGGCCCAATGAAAGACGGGATAAGTGTGTTAGAAGATCAACAGAGTTCCTTGCTTACAGTGATATCCTTGGTGCCACATTAACCTCAGTTATTACTATTTGTTCAGTGTTGACAGCTGTCATCTTTGCTGTATTTGTTAAATTTTCTGGAACTGTTATTGTTAAGGCCAACAACAGAAACATCAGCTACTTACTTCTCTTTGCCCTTCTAATTTGTTTCCTCTGCTCTTTGATCTTTATTGGATACCCTGACAAATACACTTGTATATTTCGTCAAATTGTGTTTGGTGTAGCCTTCGTGCTTTCTGTTTCTTGTGTACTGGCAAAAACCATTATGGTTGTTGTAGCTTTCAATGCAACAAAGCCAAACAGTAACTTGCAAAGGTGGATGGTGCCACAGCTTCCATATGCAATTGTATCATTGTGCACATTTGCACAGTTTATTCTCTGTGCTACGTGGATAATATGCTCGCCCCCATTCCCAGAGCAGAACTTTAAACTCCAAGCAGGGATAATAGTTTTTCAATGCAATGAAGGTTCAGTCATTGCATTTTGGTTTATGCTTGGATTTATGGCCCTACTAGCTGGTGTCTGTTTTATAATAGCATTTTTGTCACGAAACCTACCAGATAGCTTTAATGAGGCCAAGTTCATTGCATTCAGTATGATTGTATTTGTTAGTGTCTGGCTGGCCTTCATCCCAGCCTACCTCAGTACTAAGGGCAAATATACAGTGGCTGTAGAAATATTTGCTATTCTTGCTTCTAGTGCAGGTTTGCTTCTTTGTATATACTTCCCAAAATGTTACATTATTTTACTGAGGCCAGATATGAATACAAAAGAATATATAATGGGCAGAGGTCAAAGAAAAATGTAA

>annecV2R358

ATGTGGAACAATGGTTATACACAGCCTCCCAGATCAGTCTGCAGTGAAAGCTGCCTCCCAGGGTTTAGAAAATCTGCCCAGAATGGGAAACCTGAATGCTGTTATGACTGTGTACCATGTTCTGAAGGAGAAATATCCAACCAGAGTGATTCCAATATCTGCCTGGCATGTCTTCCGGAAGATTGGCCCAATGAAAGACGGGATAAGTGTGTTAGAAGATCAACAGAGTTCCTTGCTTACAGTGATATCCTTGGTGCCACATTAACCTCAGTTATTACTATTTGTTCAGTGTTGACAGCTGTCATCTTTGCTGTATTTGTTAAATTTTCTGGAACTGTTATTGTTAAGGCCAACAACAGAAACATCAGCTACTTACTTCTCTTTGCCCTTCTAATTTGTTTCCTCTGCTCTTTGATCTTTATTGGATACCCTGACAAATACACTTGTATATTTCGTCAAATTGTGTTTGGTGTAGCCTTCGTGCTTTCTGTTTCTTGTGTACTGGCAAAAACCATTATGGTTGTTGTAGCTTTCAATGCAACAAAGCCAAACAGTAACTTGCAAAGGTGGATGGTGCCACAGCTTCCATATGCAATTGTATCATTGTGCACATTTGCACAGTTTATTCTCTGTGCTACGTGGATAATATGCTCGCCCCCATTCCCAGAGCAGAACTTTAAATTCCAGGCAGGGATAATAGTTTTTGAATGCAATGAAGGTTCAGTCATTGCATTTTGGTTTATGCTTGGATTTATGGCCCTACTAGCTGGTGTCTGTTTTATAATAGCATTTTTGTCACGAAACCTACCAGATAGCTTTAATGAGGCCAAGTTCATTGCATTCAGTATGATTGTATTTGTTAGTGTCTGGCTGGCCTTCATCCCAGCCTACCTCAGTACTAAAGGCAAATATACAGTGGCTGTAGAAATATTTGCTATTATTGCTTCTAGTGCAGGTTTGCTTCTTTGTATATACTTTCCAAAATGTTACATTATTTTACTGAGGCCAGATATGAATACAAAAGAATATATAATGGGCAGAGGTCAAAGAAAAATGTAA

>annecV2R360

ATGTGGAACAATGGTTATACACAGCCTCCCAGATCAGTCTGCAGTGAAAGCTGCCTCCCAGGGTTTAGAAAATCTGCCCAGAATGGGAAACCTGAATGCTGTTATGACTGTGTACCATGTTCTGAAGGAGAAATATCCAACCAGAGTGATTCCAATATCTGCCTGGCATGTCTTCCGGAAGATTGGCCCAATGAAAGACGGGATAAGTGTGTTAGAAGATCAACAGAGTTCCTTGCTTACAGTGATATCCTTGGTGCCACATTAACCTCAGTTATTACTATTTGTTCAGTGTTGACAGCTGTCATCTTTGCTGTATTTGTTAAATTTTCTGGAACTGTTATTGTTAAGGCCAACAACAGAAACATCAGCTACTTACTTCTCTTTGCCCTTCTAATTTGTTTCCTCTGCTCTTTGATCTTTATTGGATACCCTGACAAATACACTTGTATATTTCGTCAAATTGTGTTTGGTGTAGCCTTCGTGCTTTCTGTTTCTTGTGTACTGGCAAAAACCATTATGGTTGTTGTAGCTTTCAATGCAACAAAGCCAAACAGTAACTTGCAAAGGTGGATGGTGCCACAGCTTCCATATGCAATTGTATCATTGTGCACATTTGCACAGTTTATTCTCTGTGCTACGTGGATAATATGCTCGCCCCCATTCCCAGAGCAGAACTTTAAACTCCAAGCAGGGATAATAGTTTTTCAATGCAATGAAGGTTCAGTCATTGCATTTTGGTTTATGCTTGGATTTATGGCCCTACTAGCTGGTGTCTGTTTTATAATAGCATTTTTGTCACGAAACCTACCAGATAGCTTTAATGAGGCCAAGTTCATTGCATTCAGTATGATTGTATTTGTTAGTGTCTGGCTGGCCTTCATCCCAGCCTACCTCAGTACTAAGGGCAAATATACAGTGGCTGTAGAAATATTTGCTATTCTTGCTTCTAGTGCAGGTTTGCTTCTTTGTATATACTTCCCAAAATGTTACATTATTTTACTGAGGCCAGATATGAATACAAAAGAATATATAATGGGCAGAGGTCAAAGAAAAATGTAA

>annecV2R363

ATGTGGAACAATGGTTATACACAGCCTCCCAGATCAGTCTGCAGTGAAAGCTGCCTCCCAGGGTTTAGAAAATCTGCCCAGAATGGGAAACCTGAATGCTGTTATGACTGTGTACCATGTTCTGAAGGAGAAATATCCAACCAGAGTGATTCCAATATCTGCCTGGTATGTCTTCCAGAAGATTGGCCCAATGAAAGACGGGATAAGTGTGTTAGAAGATCAACAGAGTTCCTTGCTTACAGTGATATCCTTGGTGCCACATTAACCTCAGTTATTACTATTTGTTCAGTGTTGACAGCTGTCATCTTTGCTGTATTTGTTAAATTTTCTGGAACTGTTATTGTTAAGGCCAACAACAGAAACATCAGCTACTTACTTCTCTTTGCCCTTCTAATTTGTTTCCTCTGCTCTTTGATCTTTATTGGATACCCTGACAAATACACTTGTATATTTCGTCAAATTGTGTTTGGTGTAGCCTTCGTGCTTTCTGTTTCTTGTGTACTGGCAAAAACCATTATGGTTGTTGTAGCTTTCAATGCAACAAAGCCAAACAGTAACTTGCAAAGGTGGATGGTGCCACAGCTTCCATATGCAATTGTATCATTGTGCACATTTGCACAGTTTATTCTCTGTGCTACGTGGATAATATGCTCGCCCCCATTCCCAGAGCAGAACTTTAAATTCCAGGCAGGGATAATAGTTTTTGAATGCAATGAAGGTTCAGTCATTGCATTTTGGTTTATGCTTGGATTTATGGCCCTACTAGCTGGTGTCTGTTTTATAATAGCATTTTTGTCACGAAACCTACCAGATAGCTTTAATGAGGCCAAGTTCATTGCATTCAGTATGATTGTATTTGTTAGTGTCTGGCTGGCCTTCATCCCAGCCTACCTCAGTACTAAAGGCAAATATACAGTGGCTGTAGAAATATTTGCTATTATTGCTTCTAGTGCAGGTTTGCTTCTTTGTATATACTTTCCAAAATGTTACATTATTTTACTGAGGCCAGATATGAATACAAAAGAATATATAATGGGCAGAGGTCAAAGAAAAATGTAA

>annecV2R528

ATGTGGAACCAGAACAATAAACAGATTCCTAGATCTGTCTGCAGTGAAAGATGCATCCCAGGATATAGAAAGGTTGCTCAGAATGGTAGACCTATTTGCTGCTATGATTGTGTCCAGTGTGCTGAAGGGGAAATCTCCAATTATAGTGATTCCACTAATTGCCTGTTGTGTTCATCTGAAGAATGGCCCAATGAAAGAAGGGACAAGTGTATCCAAAGAGCTCTACAGTTTCTCTCCTACAGTGAGCCCTTGGGTCTAGCTTTGGCTTTCATTGCAACAATAAGTACTCTGTTAACAGGAACTGTACAAATTATATTTATCAAGTATAGTGGAACTGCTGTTGTGAAGGCTAACAACAAAAACCTCAGCTACCTGCTTCTGGTTTCACTTATGCTATGTTTCTTGAGCTCTATGATTTTTATTGGACAACCTACAAGAGTTACCTGCAAGCTGTCTCAGTCAATGTTTGGAATAACTTTTGTTCTTTCAGTGTCGTCTGTGCTGGCTAAGACCATTACAGTAGTAGTTGCTTTTAATGCAACCAAGCCAAGTAGCAATTTGAGGAAATGCTTAGGACTACAGCTGCCTTACACTGTGTTATCCCTGAGTACTGTTGTTCAAGTCATTTTATGTGCTGTCTGGAACATTTGTGCACCTCCATTTCCAGAAAGAAACTACAGAACCCAAACAGGGCTTATAGTAATTGAATGCAATGAGGGTTCCTCAATTGCATTTTGGTGTATGTTAGGATATATTGGCTTGCTTGCTAGTGTTTGTTTTGTAGTAGCTTTCTTGTCAAGAAACCTGCCTGACAGTTTTAATGAGGCCAAGTTTATTACCTTCAGCATGGTGGTATTTGTTAGTGTGTGGCTGGCATTTATTCCAGCCTACCTAAGTACTAAGGGCAAATATACAGTAGCTGTTGAGGTATTTGCTATTGTTGCCTCTGCTTTAGGTTTGCTGACCTGCATATTCCTCCCAAAATGTTACATAATTGTTCTAAAACCTGATATTAACAAAAAAGAATATTTAACAAGATAA

>annecV2R701

ATGAAACCTCTAGGCCAGAGCCTGAATTTTACAAATAAAGATCTTATATTCAATGGCCACCAGAAACAGGCTCCTAGATCTGTGTGCAGTGAAACATGCCTTCCAGGCTTTAGAAAAGCTATTCAGAATGGGAGACCAGCATGTTGTTTTGACTGTGTTCTCTGTGCCGATGGGGAAATTGCCAATTACAGTGATTCTAAAGATTGCTGGACTTGTCCACTGGAAGACTGGCCAAATGCAAGAAAGGATAAATGTATTCTAAGACCTGTGGAGTTCCTGTCTTATAATGATGTCTTGGGAGCAATTTTAGCATGTACAGCATTTGTATTTTCATTATTATCAATTGCAGTCTTACTTATCTTTGTAAAATTTAGTGAAACGCCTGTTGTCAAAGCCAATAACCGAAATATTAGCTATCTCCTTCTAGCTGGACTTTTGCTGTGCTTTCTGAGTTCATTACTTTTTATTGGGCAGCCAAGCCAAGCTTCCTGCAGGCTACGTCAAACTCTGTTTGGTGTAATCTTTGTCCTTTCTGTGTCCTGTGTATTGGCCAAGACCATTATGGTAGTTGTCGCTTTCAGAGCTACAATGCCAAACAGTTCCATGAGAAAATGGGTTGGACCACACCTTTCTTATGTTATAGTGTCCAGTTGTACAAGTGTCCAGGTTGTTATCTGTGCAAGCTGGGTGACATTTTCTCCTCCTTTTTATGAGCATAACTATGCATCAAAACCAGGAATTAAAATTATTGAATGCAATGAAGGGTCTGCCATTGCATTTTGGTTCATGCTGGGGTACATGGGACTGTTATCAATTATATGCTTTATTGTAGCATTTTTGTCAAGACAACTTCCTGATAGCTTTAACGAGGCTAAGTTTATCACCTTCAGCATGGTTGTGTTTGCTAGTACTTGGCTAGCCTTCATTCCAGCCTACCTCAGTACCAAGGGTAAATTCATGGTGGTTGTAGAAATATTTGCAATCATTTCTTCTGGTGTTGGCCTGCTTTTCTGCATATTTATTCCAAAATGTTTTATTATTGTCCTCAGACCAGAAATGAATTCAAAAGACTACTTAAGGGGTAAAACAGCACTTGACTAA

>annecV2R712

ATGTGGAACAGTCAACAAACACAGGCTCCACGATCTGTCTGCAGTGAAACCTGTTCTCCTGGATTCAGAAAGGTTGCTCAGAGAGAAAGACCTCTTTGTTGTTTTGACTGTGTTTGGTGTCCTGAAGGAGAAATTTCCAACCAGAGTGATTCCAAGGACTGTCTCAGTTGTCCAAAACATGAGTGGCCAAGGGCAAGAAAAGATAAATGCATTCAGAGATCAATCGACTTTCTTTCATTTTATGATGCTCTTGGTACAAGTTTGGCAGTGATTGCCATTGTTAGTTTTATTCTGGTGGCACTTATATTTATTACTTTTGTGAAATTTCATGAAACTCCCCTAGTTAAAGCCAATAATCGGAATCTAAGTTTTATACTTCTGGCAGCCCTTATGCTCTGTTCTCTGTGTCCTTTGATGTTTATTGGATATCCTACCATAGCTACTTGTAGATTTCGTCAAGCTGTGTTTGGTATAACATTTGTACTTTCTGTTTCATGTGTTTTGATGAAAACCATTATGGTAGTCATTGCCTTCAAGGCAGCAAAACCAGGCAGCACCTTGTTAACATGGGTGGGACCACAGCTTACATATACTGCACTGAGCCTGTGCACACTTATTCAGATTATTATATGTATTACTTGGATATTGGTTTCACCCCCGTTCCCAGAGCTAAACTACAAAATCCAGCCTGGAGTGATAGTAGTTGAATGTAATGAAGGTTCAACCACTGCATTTTGGTTCATGTTGGGATACCTTGGCATTCTGTCAGGAGTTTGTTTTATAGTAGCTTTCTTGTCTAGGAAATTACCTGATAGCTTTAATGAAGCCTTGCATATTACATTCAGCATGGTTGTATTTATCAGTGTGTGGCTTGCCTTTATTCCAGCTTATTACAGTACAAAGGGAAAGTATATGGTAGCAGTAGAAATATTTGCAATTATTTGTTCTGTTGCTGGATTGGTTGGCTGCATTTTTTTCCCAAAGTGTTTTATCATTTTGATGAGACCTGATAAGAATACAAAACAATATATTATGAACAGAGGCTCCCGGGGTGACTGA
